# Natriuretic peptides and integrated risk assessment for cardiovascular disease: an individual-participant-data meta-analysis

**DOI:** 10.1016/S2213-8587(16)30196-6

**Published:** 2016-10

**Authors:** Natriuretic Peptides Studies Collaboration

## Abstract

**Background:**

Guidelines for primary prevention of cardiovascular diseases focus on prediction of coronary heart disease and stroke. We assessed whether or not measurement of N-terminal-pro-B-type natriuretic peptide (NT-proBNP) concentration could enable a more integrated approach than at present by predicting heart failure and enhancing coronary heart disease and stroke risk assessment.

**Methods:**

In this individual-participant-data meta-analysis, we generated and harmonised individual-participant data from relevant prospective studies via both de-novo NT-proBNP concentration measurement of stored samples and collection of data from studies identified through a systematic search of the literature (PubMed, Scientific Citation Index Expanded, and Embase) for articles published up to Sept 4, 2014, using search terms related to natriuretic peptide family members and the primary outcomes, with no language restrictions. We calculated risk ratios and measures of risk discrimination and reclassification across predicted 10 year risk categories (ie, <5%, 5% to <7·5%, and ≥7·5%), adding assessment of NT-proBNP concentration to that of conventional risk factors (ie, age, sex, smoking status, systolic blood pressure, history of diabetes, and total and HDL cholesterol concentrations). Primary outcomes were the combination of coronary heart disease and stroke, and the combination of coronary heart disease, stroke, and heart failure.

**Findings:**

We recorded 5500 coronary heart disease, 4002 stroke, and 2212 heart failure outcomes among 95 617 participants without a history of cardiovascular disease in 40 prospective studies. Risk ratios (for a comparison of the top third *vs* bottom third of NT-proBNP concentrations, adjusted for conventional risk factors) were 1·76 (95% CI 1·56–1·98) for the combination of coronary heart disease and stroke and 2·00 (1·77–2·26) for the combination of coronary heart disease, stroke, and heart failure. Addition of information about NT-proBNP concentration to a model containing conventional risk factors was associated with a C-index increase of 0·012 (0·010–0·014) and a net reclassification improvement of 0·027 (0·019–0·036) for the combination of coronary heart disease and stroke and a C-index increase of 0·019 (0·016–0·022) and a net reclassification improvement of 0·028 (0·019–0·038) for the combination of coronary heart disease, stroke, and heart failure.

**Interpretation:**

In people without baseline cardiovascular disease, NT-proBNP concentration assessment strongly predicted first-onset heart failure and augmented coronary heart disease and stroke prediction, suggesting that NT-proBNP concentration assessment could be used to integrate heart failure into cardiovascular disease primary prevention.

**Funding:**

British Heart Foundation, Austrian Science Fund, UK Medical Research Council, National Institute for Health Research, European Research Council, and European Commission Framework Programme 7.

## Introduction

Cardiovascular disease guidelines recommend strategies that predict and prevent composite endpoints for coronary heart disease and stroke.^1^, ^2^, ^3^, ^4^ A rationale for this combined approach is to enhance efficiency of cardiovascular disease screening by capitalising on shared risk factors and preventive interventions, even though coronary heart disease and stroke are aetiologically distinct. Such a rationale could be extended to heart failure. The age-specific incidence of heart failure is increasing; it is a common initial presentation of cardiovascular disease.^5^ Furthermore, statins and antihypertensive treatments might, in addition to their benefits for primary prevention of coronary heart disease and stroke, be effective at reducing the risk of new-onset heart failure.^6^, ^7^ Practical advantages of a strategy that integrates heart failure prediction into cardiovascular disease risk assessment could exist since coronary heart disease and stroke risk assessment is already widespread, whereas primary prevention of heart failure is not addressed by current guidelines.^8^, ^9^

One approach that could enable such an integrated strategy is measurement of soluble natriuretic peptides. These molecules play important roles in regulation of blood pressure, blood volume, and sodium balance.^10^ Assessment of circulating B-type natriuretic peptide concentration and its more stable by-product N-terminal-pro-B-type natriuretic peptide (NT-proBNP) is recommended by guidelines for diagnosis and management of patients with heart failure.^8^, ^9^ As natriuretic peptides are markers of vascular remodelling, their measurement could also serve as an adjunct in prediction of first-ever coronary heart disease and stroke outcomes.^11^ However, to what extent assessment of natriuretic peptides can predict first-onset heart failure outcomes or improve prediction of coronary heart disease and stroke in people without known cardiovascular disease is uncertain.^12^, ^13^, ^14^, ^15^, ^16^ To address these questions, we established the Natriuretic Peptides Studies Collaboration, an international consortium of individual-participant data from individuals without a history of cardiovascular disease at baseline.

**Research in context**

**Evidence before this study**

We hypothesised that integrated cardiovascular disease risk assessment strategies could be extended to primary prevention of heart failure through measurement of N-terminal-pro-B-type natriuretic peptide (NT-proBNP) concentration. In a systematic review of the published literature (searches of PubMed, Scientific Citation Index Expanded, and Embase for relevant articles published up to Sept 4, 2014, using search terms related to natriuretic peptide family members and the primary outcomes, with no language restrictions), we identified 33 relevant prospective studies of natriuretic peptides and incident coronary heart disease, stroke, or heart failure outcomes. We attempted a synthesis of these results in a previous literature-based review, but we found that using published results was insufficiently powered or detailed or both to enable reliable assessment of whether or not NT-proBNP concentration measurement could augment cardiovascular disease risk assessment for coronary heart disease and stroke, and investigators of only few population-based prospective studies reported on associations between NT-proBNP concentration and first-onset heart failure.

**Added value of this study**

The Natriuretic Peptides Studies Collaboration involved new NT-proBNP concentration measurements in eight prospective studies as well as collation and harmonisation of individual-participant data from a further 32 relevant prospective cohorts identified by an updated systematic review. This effort enabled a detailed and standardised analysis of primary data for 95 617 participants without a history of cardiovascular disease recruited into 40 prospective studies in 12 different countries. The key added value of this collaboration is its ability to derive valid and new insights by combination of individual-participant data, information about various established and emerging risk factors, extended follow-up, breadth of cardiovascular disease outcomes recorded (eg, fatal and non-fatal heart failure, coronary heart disease, and stroke), study of several different measures of predictive ability, and generalisability to several high-income industrialised countries.

**Implications of all the available evidence**

We found that NT-proBNP concentration assessment strongly predicted first-onset heart failure and augmented coronary heart disease and stroke prediction. The incremental predictive ability of NT-proBNP concentration for coronary heart disease and stroke was moderate, but still greater than were those for HDL cholesterol or C-reactive protein concentrations. Our results have suggested that NT-proBNP concentration assessment could serve as a multipurpose biomarker in new approaches that integrate heart failure into cardiovascular disease primary prevention.

## Methods

### Data sources

Using two complementary approaches, we generated, collated, and harmonised individual-participant-level data from relevant prospective cohorts. First, de-novo NT-proBNP concentration measurements of stored samples were done by technicians masked to case-control status for some studies using the Elecsys2010 electrochemiluminescence method (proBNP Generation II; Roche, Burgess Hill, UK; appendix p 4). Second, we sought individual-participant data from relevant prospective studies identified through systematic searches of the published literature (PubMed, Scientific Citation Index Expanded, and Embase) for articles published up to Sept 4, 2014, using search terms related to natriuretic peptide family members and the primary outcomes, with no language restrictions (appendix p 7). We also scanned reference lists of identified articles for additional relevant studies. Studies were eligible if they had assayed NT-proBNP or B-type natriuretic peptide (BNP) concentration; recorded baseline information about age, sex, smoking status, systolic blood pressure, history of diabetes, and total and HDL cholesterol concentration (conventional risk factors); included participants without a known history of cardiovascular disease (ie, coronary heart disease, stroke, transient ischaemic attack, peripheral vascular disease, cardiovascular surgery, pulmonary heart disease, atrial fibrillation, or heart failure) at entry into the study; and recorded cause-specific deaths or major cardiovascular morbidity (non-fatal myocardial infarction, stroke, or heart failure) using well defined criteria over at least 1 year of follow-up.

The appendix (p 4) provides details of the methods used to collect and harmonise data. Contributing studies classified deaths according to the primary cause (or, in its absence, the underlying cause) on the basis of International Classification of Diseases coding, revisions 8–10, to at least three digits, or according to study-specific classification systems. We based ascertainment of fatal outcomes on death certificates, supplemented in 26 cohorts by additional data, and of non-fatal outcomes on WHO (or similar) criteria for myocardial infarction and on clinical and imaging features for stroke and heart failure (appendix p 18). This Article follows Preferred Reporting Items for Systematic Reviews and Meta-Analyses for Individual Patient Data reporting (appendix pp 8–11).^17^ The study was designed and done by the Natriuretic Peptides Studies Collaboration's independent coordinating centre and approved by the Cambridgeshire Ethics Review Committee.

### Data analysis

The analysis involved three inter-related components. First, we characterised cross-sectional associations of NT-proBNP concentration with established and emerging risk factors. Second, we assessed associations of NT-proBNP concentration with first-onset coronary heart disease, stroke, and heart failure, singly and in combination. Third, we quantified the incremental predictive value of assessment of NT-proBNP concentration in addition to conventional risk factors for major cardiovascular disease outcomes.

We focused the principal analyses on NT-proBNP concentration data because NT-proBNP is a more stable analyte than is BNP and encompassed more than 95% of the data in the collaboration (reserving the sparse BNP data for supplementary analyses). We defined two primary outcomes: 1) a combination of coronary heart disease (defined as fatal or non-fatal myocardial infarction) and stroke and 2) a combination of coronary heart disease, stroke, and heart failure. Participants contributed only the first cardiovascular disease outcome (whether non-fatal or fatal) recorded during follow-up (ie, we did not include deaths preceded by non-fatal cardiovascular disease events). Secondary outcomes were the component cardiovascular disease outcomes (ie, coronary heart disease, stroke, and heart failure) and the aggregate of death due to additional cardiovascular disease outcomes (ie, cardiac arrhythmia, hypertensive disease, pulmonary embolism, complications and ill defined descriptions of heart disease, sudden death, aortic aneurysms, and peripheral vascular disease). We censored outcomes if a participant was lost to follow-up, died from causes other than cardiovascular diseases, or reached the end of the follow-up period.

We calculated hazard ratios from prospective studies with Cox proportional hazard regression models, stratified by sex, using time-on-study as a timescale. We assessed the proportional hazards assumption, which was satisfied, as previously described.^18^ Analyses of case-cohort data involved Prentice weights and robust SEs.^19^ We calculated odds ratios from nested case-control studies using logistic regression models. We assumed hazard and odds ratios to represent the same relative risk, collectively describing them as risk ratios. We calculated risk ratios for a comparison of individuals in the top third with those in the bottom third of baseline NT-proBNP values using a two-stage approach, with estimates calculated separately within each study before pooling across studies with multivariate random-effects meta-analysis.^18^ To characterise shapes of associations, we calculated pooled risk ratios within overall tenths of NT-proBNP concentration and plotted them against the pooled geometric mean of NT-proBNP concentration within each tenth. We adjusted risk ratios for baseline levels of conventional risk factors. We investigated effect modification by study-level and individual characteristics with meta-regression and formal tests of interaction.^18^ We assessed between-study heterogeneity with the *I*^2^ statistic.^20^

We developed cardiovascular disease risk prediction models containing information about conventional risk factors with or without NT-proBNP concentration only in cohort and case-cohort studies and quantified improvements in predictive ability using measures of risk discrimination and reclassification.^21^, ^22^ We calculated C-indices and C-index changes within each study before pooling results weighted by the number of outcomes contributed. We calculated measures of risk reclassification (ie, integrated discrimination improvement and categorical and continuous net reclassification improvement) using data from studies in which both fatal and non-fatal events had been recorded.^21^ We examined categorical net reclassification of participants across predicted 10 year risk categories using cutoffs defined by the American College of Cardiology (ACC) and American Heart Association (AHA) 2013 (ie, <5%, 5% to <7·5%, and ≥7·5%),^1^ National Institute of Health and Care Excellence 2014,^4^ American College of Cardiology Foundation and American Heart Association 2010,^3^ and European Society of Cardiology 2016 guidelines.^2^ We log-transformed NT-proBNP concentration and modelled it using both linear and quadratic terms (with similar approaches used for the analysis of HDL cholesterol and C-reactive protein [CRP] concentration). We did analyses using Stata software, version 12.1. All p values are two sided. The appendix (pp 5–6) provides further details of the analytical methods used.

### Role of the funding source

The funders of the study had no role in study design, data collection, data analysis, data interpretation, or writing of the report. PWi, JD, and EDA had full access to all the data in the study and had final responsibility for the decision to submit for publication.

## Results

Measurement of stored samples from 7129 participants (including 1173 incident cardiovascular disease cases) was done for eight prospective studies (the Reykjavik Offspring Study,^23^ the Northern Sweden Health and Disease Study,^24^ the Bruneck Study,^25^ and five cohorts contributing to the DAN-MONICA study;^26^ appendix p 3). We sought individual-participant data from 33 relevant prospective studies. Only one potentially relevant study^27^ (comprising <3% of the cardiovascular disease outcomes) was unable to contribute data, yielding a total of 40 contributing prospective studies from 12 countries (of which 30 had been analysed as cohort studies, eight as case-cohort studies, and two as nested case-control studies) and 95 617 participants without a history of cardiovascular disease. Details of the 40 contributing studies are provided in the appendix (pp 12, 17–20).^12^, ^13^, ^14^, ^15^, ^16^, ^23^, ^24^, ^25^, ^26^, ^28^, ^29^, ^30^, ^31^, ^32^, ^33^, ^34^, ^35^, ^36^, ^37^, ^38^, ^39^, ^40^, ^41^, ^42^, ^43^, ^44^, ^45^, ^46^, ^47^, ^48^, ^49^, ^50^, ^51^

48 528 (51%) of participants were women and 61 451 (64%) were from Europe, and mean age at baseline was 61 years (SD 10). Median NT-proBNP concentration was 64 pg/mL (IQR 30–135; appendix pp 19–20, 27). NT-proBNP concentrations were approximately linearly associated with BNP concentrations across the range of values (appendix p 28). NT-proBNP and BNP concentrations increased with age and were higher in women, but were only weakly associated with several other characteristics, including ethnicity, history of hypertension, use of antihypertensive medication, systolic blood pressure, total and HDL cholesterol concentration, and estimated glomerular filtration rate (appendix pp 21, 22, 29).

**Figure 1:**
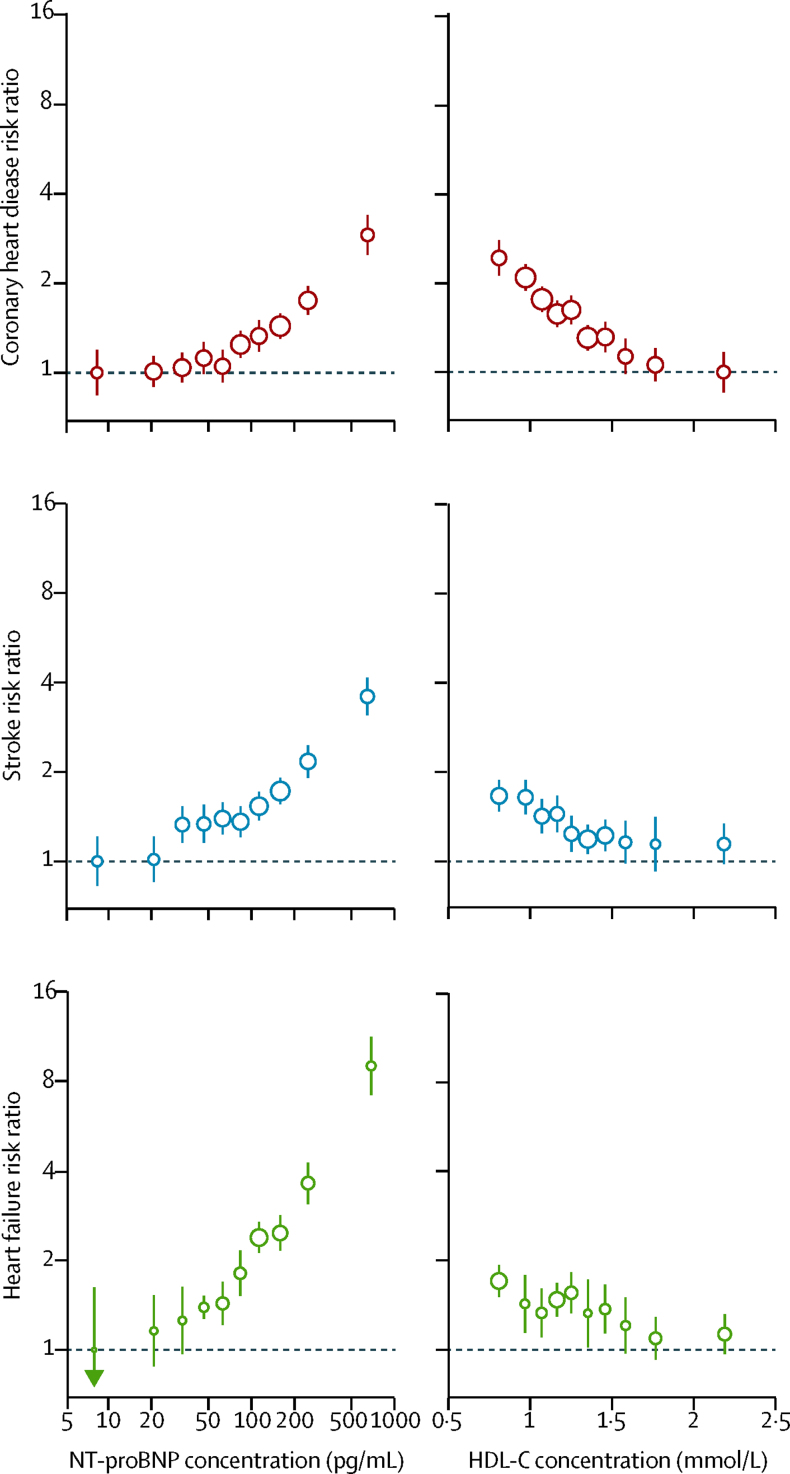
Associations of NT-proBNP and HDL-C concentrations with first-onset coronary heart disease, stroke, and heart failure Risk ratios adjusted for age, smoking status, history of diabetes, systolic blood pressure, and total cholesterol and HDL-C concentration (HDL-C concentration only for NT-proBNP concentration analysis) and stratified by sex. Analyses involved 4716 coronary heart disease outcomes (from 34 cohorts), 3768 stroke outcomes (from 30 cohorts), and 2021 heart failure outcomes (from 16 cohorts). The size of the circles is proportional to the inverse of the variance of the respective estimate. Error bars are 95% CIs, estimated from floated variances. HDL-C=HDL cholesterol. NT-proBNP=N-terminal-pro-B-type natriuretic peptide.

**Figure 2:**
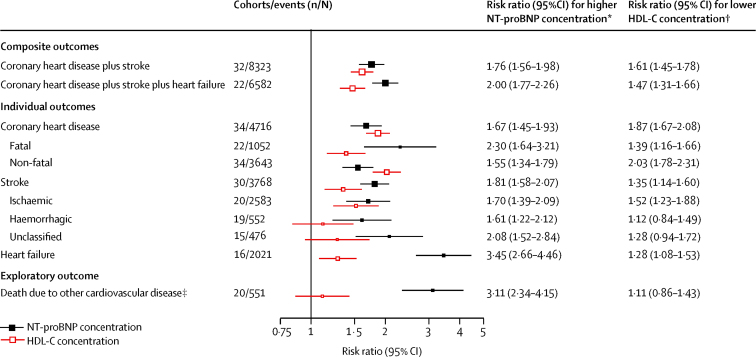
Associations of NT-proBNP and HDL-C concentrations with several incident first-onset cardiovascular outcomes Risk ratios adjusted for age, smoking status, history of diabetes, systolic blood pressure, and total cholesterol and HDL-C concentration (HDL-C concentration only for NT-proBNP concentration analysis) and stratified by sex. HDL-C=HDL cholesterol. NT-proBNP=N-terminal-pro-B-type natriuretic peptide. *Top versus bottom third of NT-proBNP concentration. †Bottom versus top third of HDL-C concentration. ‡Subsumes deaths due to cardiac arrhythmia, hypertensive disease, pulmonary embolism, complications and ill defined descriptions of heart disease, sudden death, aortic aneurysms, and peripheral vascular disease.

During 809 525 person-years at risk (median follow-up 7·8 years [IQR 5·2–11·8]), 5500 coronary heart disease, 4002 stroke, and 2212 heart failure outcomes occurred. NT-proBNP concentration was non-linearly associated with the risk of each of these diseases (figure 1). Risk ratios (top third *vs* bottom third of NT-proBNP concentration) adjusted for conventional risk factors were 1·76 (95% CI 1·56–1·98) for the combination of coronary heart disease and stroke; 2·00 (1·77–2·26) for the combination of coronary heart disease, stroke, and heart failure; 1·67 (1·45–1·93) for coronary heart disease; 1·81 (1·58–2·07) for stroke; 3·45 (2·66–4·46) for heart failure; and 3·11 (2·34–4·15) for cardiovascular disease deaths due to additional causes (figure 2; appendix p 30). Risk ratios were somewhat higher for fatal than for non-fatal coronary heart disease (p<0·0001), but similar for ischaemic and haemorrhagic stroke (p=0·44). In the same participants, corresponding risk ratios with lower HDL cholesterol concentration were 1·61 (1·45–1·78) for the combination of coronary heart disease and stroke and 1·47 (1·31–1·66) for the combination of coronary heart disease, stroke, and heart failure.

Risk ratios for NT-proBNP concentration did not materially change with further adjustment for body-mass index or estimated glomerular filtration rate, but they reduced somewhat with adjustment for CRP concentration (appendix p 23). Risk ratios for heart failure were higher in men than in women (4·25 *vs* 2·44; p<0·0001), in participants with a low body-mass index than a high body-mass index (3·61 *vs* 2·76; p=0·0004), and in studies that had stored samples for 10 years or fewer before analysis than longer than 10 years (6·20 *vs* 2·68; p=0·0018; appendix p 31–32). Otherwise, risk ratios did not vary substantially with levels of conventional risk factors or in other clinically relevant subgroups (appendix pp 31–32). We observed qualitatively similar findings in analyses that defined thirds separately for men and women, excluded people with high baseline concentrations of NT-proBNP, excluded the initial 5 years of follow-up, and were restricted to studies recording both fatal and non-fatal outcomes (appendix p 33). Similar findings were also noted in analyses that compared studies grouped by NT-proBNP concentration assay type or generation (appendix p 32), compared studies with different lengths of follow-up (appendix p 34), used per one SD higher log NT-proBNP concentration (appendix p 24), and focused on fatal outcomes only (appendix p 35). In analyses of 15 909 participants for coronary heart disease and stroke and 12 202 participants for heart failure from seven studies with available information about BNP concentration,^28^, ^29^, ^30^, ^31^, ^32^, ^33^, ^34^ risk ratios for coronary heart disease, stroke, and heart failure observed with BNP concentration were weaker than were those observed with NT-proBNP concentration (appendix p 25). We noted moderate heterogeneity of risk ratios across studies (appendix p 30). *I*^2^ values were 45% for coronary heart disease, 23% for stroke, and 54% for heart failure.

**Figure 3:**
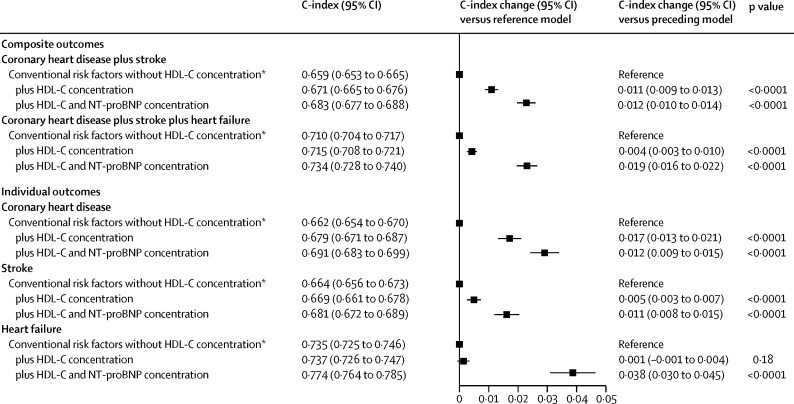
Improvement in risk discrimination for first-onset individual and composite cardiovascular disease outcomes by addition of information about NT-proBNP concentration compared with that about HDL-C concentration Analyses involved 8323 outcomes for the combination of coronary heart disease plus stroke (from 32 cohorts), 6582 outcomes for the combination of coronary heart disease plus stroke plus heart failure (from 22 cohorts), 4552 coronary heart disease outcomes (from 32 cohorts), 3768 stroke outcomes (from 30 cohorts), and 2021 heart failure outcomes (from 16 cohorts). HDL-C=HDL cholesterol. NT-proBNP=N-terminal-pro-B-type natriuretic peptide. *The reference model included information about age, sex, smoking, systolic blood pressure, history of diabetes, and concentration of total cholesterol.

**Figure 4:**
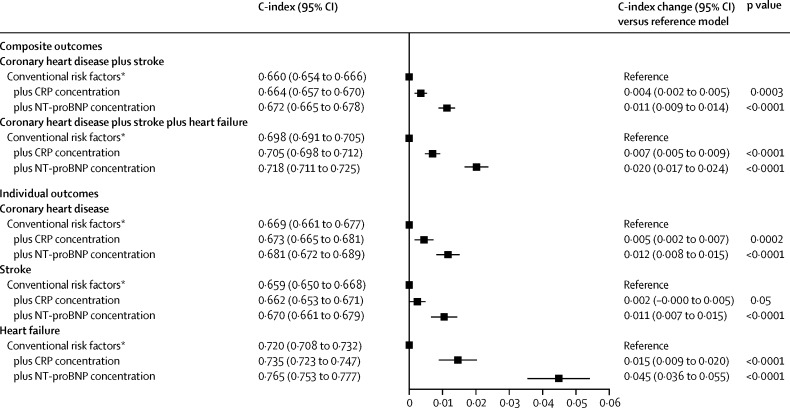
Improvement in risk discrimination for first-onset individual and composite cardiovascular outcomes by addition of information about CRP and NT-proBNP concentration to a model with conventional risk factors Analyses involved 7618 outcomes for the combination of coronary heart disease plus stroke (from 28 cohorts), 5492 outcomes for the combination of coronary heart disease plus stroke plus heart failure (from 18 cohorts), 4120 coronary heart disease outcomes (from 27 cohorts), 3487 stroke outcomes (from 26 cohorts), and 1606 heart failure outcomes (from 13 cohorts). CRP=C-reactive protein. NT-proBNP=N-terminal-pro-B-type natriuretic peptide. *The reference model included information about age, sex, smoking, systolic blood pressure, history of diabetes, and concentrations of total and HDL cholesterol.

After addition of NT-proBNP concentration to a model with conventional risk factors only, the C-index increased by 0·012 (95% CI 0·010–0·014) for the combination of coronary heart disease and stroke; 0·019 (0·016–0·022) for the combination of coronary heart disease, stroke, and heart failure; 0·012 (0·009–0·015) for coronary heart disease; 0·011 (0·008–0·015) for stroke; and 0·038 (0·030–0·045) for heart failure (figure 3; appendix pp 36–37). Overall net reclassification improvements for NT-proBNP concentration across predicted 10 year risk categories defined by the 2013 ACC and AHA guidelines^1^ were 0·027 (0·019–0·036) for the combination of coronary heart disease and stroke and 0·028 (0·019–0·038) for the combination of coronary heart disease, stroke, and heart failure (table). Continuous net reclassification improvements were 0·154 (0·111–0·198) for the combination of coronary heart disease and stroke and 0·198 (0·162–0·234) for the combination of coronary heart disease, stroke, and heart failure, and integrated discrimination improvements were 0·013 (0·011–0·015) for the combination of coronary heart disease and stroke and 0·030 (0·026–0·033) for the combination of coronary heart disease, stroke, and heart failure (appendix p 26). Incremental risk prediction afforded by NT-proBNP concentration assessment was greater than that afforded by HDL cholesterol or CRP concentration assessment (figure 3, figure 4, table). NT-proBNP and CRP concentration provided essentially non-overlapping incremental risk discrimination (figure 4).

In further analyses that involved the combination of coronary heart disease, stroke, and heart failure as the outcome, improvements in C-index with NT-proBNP concentration assessment were possibly greater among older individuals and people with a history of diabetes, who used antihypertensives, who had a higher systolic blood pressure, and who had a lower total cholesterol concentration (appendix p 38). However, we did not adjust these exploratory analyses for multiple comparisons. In further sensitivity analyses, we found that C-index improvements were similar when the base model additionally included information about ethnicity and antihypertensive treatment (appendix p 39), but somewhat smaller in analyses that excluded people with high baseline concentrations of NT-proBNP or modelled NT-proBNP concentration using a prespecified cutoff value rather than continuous values (appendix p 40). Net reclassification improvements were similar or larger than were those in the main analysis when analysis involved cutoffs for clinical risk categories defined by guidelines other than the 2013 ACC and AHA guidelines^1^ (appendix p 26).

## Discussion

In this study, we found that NT-proBNP concentration assessment strongly predicted first-onset heart failure and augmented coronary heart disease and stroke prediction, suggesting that NT-proBNP concentration assessment could serve as a multipurpose biomarker in new approaches that integrate heart failure into cardiovascular disease primary prevention. A key observation was our study's demonstration of graded associations between NT-proBNP concentration and the incidence of coronary heart disease, stroke, and heart failure. The continuous nature of these associations suggests that NT-proBNP concentration measurement is potentially suitable for population-level risk assessment. We also made the surprising observation that NT-proBNP concentration predicts stroke at least as strongly as it does coronary heart disease, by contrast with the idea that NT-proBNP concentration is predominantly a coronary biomarker. The stroke associations that we noted could partly be explained by associations previously reported between NT-proBNP concentration and stroke risk factors (eg, left ventricular hypertrophy and atrial fibrillation),^15^, ^52^ but further work is needed to elucidate the common pathobiology for coronary heart disease, stroke, and heart failure reflected by preceding NT-proBNP concentration. Furthermore, we found that NT-proBNP concentration predicted deaths due to additional cardiovascular causes, such as cardiac arrhythmia and sudden death.^53^ Collectively, these results encourage evaluation of NT-proBNP concentration for prediction of an even wider range of cardiovascular disease outcomes than that we studied.

Our conclusions on the incremental predictive ability of assessment of NT-proBNP concentration were strengthened by broadly concordant results when we studied varying cardiovascular disease outcomes and used different measures of risk discrimination and reclassification. Importantly, the modest improvements that we observed in risk reclassification with NT-proBNP concentration assessment applied similarly across the absolute risk thresholds used in different clinical guidelines.^1^, ^2^, ^3^, ^4^ In particular, NT-proBNP concentration assessment improved the specificity of risk prediction by appropriately downclassifying the clinical risk category of many individuals who did not go on to develop cardiovascular disease outcomes. Hence, addition of NT-proBNP concentration measurement to cardiovascular disease risk assessment could improve targeting of preventive treatments (such as statins) and allocation of resources for detailed screening (such as comprehensive tests for heart failure at specialised cardiology clinics), as exemplified by previous natriuretic peptide-guided trials in patients with diabetes^54^ or heart failure.^55^, ^56^ Data from future studies are needed to establish the cost-effectiveness and feasibility of NT-proBNP concentration screening for prediction of first composite cardiovascular disease outcomes, analogous with previous work on left ventricular systolic dysfunction.^57^, ^58^, ^59^

To provide clinical context, we compared incremental improvements afforded by NT-proBNP concentration assessment with those afforded by HDL cholesterol, a widely used biomarker in cardiovascular disease risk assessment (this comparison is additionally relevant because HDL cholesterol concentration, like NT-proBNP concentration, is a biomarker of unknown relevance to the cause of cardiovascular disease^60^). We found that improvements in risk discrimination with NT-proBNP concentration were greater than those provided by HDL cholesterol, even though our evaluation was skewed in favour of HDL cholesterol concentration since we added HDL cholesterol concentration only to other conventional risk factors (omitting NT-proBNP concentration), whereas we added NT-proBNP concentration to conventional risk factors, including HDL cholesterol concentration. Furthermore, in a head-to-head comparison, we found that the improvement in risk discrimination with NT-proBNP concentration was about three times greater than was the improvement in risk discrimination using CRP concentration. The idea that NT-proBNP concentration captures information about non-traditional cardiovascular disease pathways^61^, ^62^ was supported by our observation that NT-proBNP concentration was uncorrelated or weakly correlated with the established and emerging risk factors that we studied.

Our study had major strengths. Because of its considerable statistical power, we could provide precise estimates, even for analyses that involved categorisation of NT-proBNP concentrations. More than 90% of the NT-proBNP concentration data in our analysis were generated with use of a common gold-standard assay. We recorded information about the incidence of various cardiovascular disease outcomes using well validated endpoint definitions. We centrally analysed individual-participant data, which were harmonised from prospective studies with extended follow-up, enabling time-to-event analyses, exclusion of people with a baseline history of cardiovascular disease (including heart failure), and adoption of a uniform approach to statistical analyses. To enhance validity further, we restricted analyses to people with complete information about a set of relevant risk factors. Our primary analysis excluded participants with a reported baseline history of heart failure and, moreover, the findings were robust to exclusion of participants with high baseline NT-proBNP concentrations. The generalisability of our findings was enhanced by inclusion of data from 12 countries and by the robustness of results to various sensitivity analyses.

Our study had potential limitations. Misclassification of heart failure outcomes could have led to underestimation of associations between NT-proBNP concentration and heart failure risk and, conversely, overestimation of associations with non-heart failure outcomes. Most of our data were derived from people of European continental ancestry. We could not compare the performance of NT-proBNP concentration with cardiac troponin, coronary calcium scoring, or other biomarkers apart from HDL cholesterol and CRP concentrations.

We conclude that assessment of NT-proBNP concentration could serve as a multipurpose biomarker in new approaches that integrate heart failure into primary prevention of cardiovascular diseases.

## Figures and Tables

**Table:** Improvement in risk classification for first-onset composite cardiovascular disease outcomes by addition of information about NT-proBNP concentration compared with that about HDL-C

	**Non-cases**	**Cases**	**Overall**
**Coronary heart disease plus stroke**			
Conventional risk factors without HDL-C concentration*	Reference	Reference	Reference
plus HDL-C concentration	0·001 (−0·003 to 0·004); p=0·70	0·008 (−0·000 to 0·016); p=0·056	0·009 (−0·000 to 0·017); p=0·056
plus HDL-C and NT-proBNP concentration	0·029 (0·025 to 0·032); p<0·0001	−0·001 (−0·009 to 0·007); p=0·79	0·027 (0·019 to 0·036); p<0·0001
**Coronary heart disease plus stroke plus heart failure**			
Conventional risk factors without HDL-C concentration*	Reference	Reference	Reference
plus HDL-C concentration	0·011 (0·008 to 0·015); p<0·0001	0·006 (−0·001 to 0·013); p=0·10	0·017 (0·009 to 0·025); p<0·0001
plus HDL-C and NT-proBNP concentration	0·036 (0·032 to 0·040); p<0·0001	−0·008 (−0·017 to 0·001); p=0·087	0·028 (0·019 to 0·038); p<0·0001

Data are categorical net reclassification improvement versus preceding model (95% CI); p value. We calculated categorical net reclassification improvement across predicted 10 year cardiovascular disease risk categories defined by the American College of Cardiology and American Heart Association 2013 guidelines.^1^ Analyses involved 4672 outcomes for the composite outcome of coronary heart disease plus stroke (from 19 cohorts) and 4071 for the composite outcome of coronary heart disease plus stroke plus heart failure (from 16 cohorts). HDL-C=HDL cholesterol. NT-proBNP=N-terminal-pro-B-type natriuretic peptide.

* The reference model included information about age, sex, smoking, systolic blood pressure, history of diabetes, and concentration of total cholesterol.
