# Supplementary material for: Natriuretic peptides and integrated risk assessment for cardiovascular disease: an individual-participant-data meta-analysis
Source: Lancet Diabetes Endocrinol. 2016 Oct;4(10):840–9. doi: 10.1016/S2213-8587(16)30196-6 (PMC5035346; doi:10.1016/S2213-8587(16)30196-6)
Supplement: Supplementary appendix [file mmc1.pdf]

THE LANCET
Diabetes & Endocrinology

Supplementary appendix

This appendix formed part of the original submission and has been peer reviewed. We post it as supplied by the authors.

Supplement to: Natriuretic Peptides Studies Collaboration. Natriuretic peptides and integrated risk assessment for cardiovascular disease: an individual-participant-data meta-analysis. *Lancet Diabetes Endocrinol* 2016; published online Sept 2. http://dx.doi.org/10.1016/S2213-8587(16)30196-6.

Natriuretic peptides and cardiovascular disease risk assessment: individual-participant data meta-analysis of 40 prospective studies

Contents list of supplementary material

Supplementary Appendices

Pages	eAppendix no.	
3-6	1	Supplementary Methods
7	2	Systematic literature search procedures
8	3	IPD Flow Diagram
9-11	4	PRISMA-IPD checklist
12	5	List of contributing studies
13-16	6	References

Supplementary Tables

Pages	eTable no.	
17	1	Study design and measurement of the 40 cohorts
18	2	Assessment of prevalent and incident cardiovascular disease
19	3	Baseline characteristics of participants
20	4	Available follow-up information in the the 40 cohorts
21	5	Cross-sectional association of NT-proBNP
22	6	Cross-sectional association of BNP
23	7	Risk ratios for individual cardiovascular outcomes using NT-proBNP
24	8	Sensitivity analyses using study-specific distribution to define thirds
25	9	Risk ratios for individual cardiovascular outcomes using BNP
26	10	Improvement in risk classification and integrated discrimination by addition of information on NT-proBNP

Supplementary Figures

Pages	eFigure no.	
27	1	Study-specific distributions of natriuretic peptides
28	2	Association between NT-proBNP and BNP levels
29	3	Cross-sectional associations of NT-proBNP
30	4	Study-specific risk ratios for individual cardiovascular outcomes
31	5	Risk ratios across clinically relevant subgroups
32	6	Risk ratios according to different study-level characteristics
33	7	Sensitivity analyses of the association of NT-proBNP with cardiovascular outcomes
34	8	Risk ratios according to lengths of follow-up.
35	9	Associations of NT-proBNP and HDL-cholesterol with fatal cardiovascular outcomes
36	10	Study-specific C-indices for the composite outcome of CHD plus stroke
37	11	Study-specific C-indices for the composite outcome of CHD plus stroke plus heart failure
38	12	Improvement in risk discrimination across clinically relevant subgroups.
39	13	Improvement in risk discrimination using a model with information on conventional risk factors, ethnicity, and anti-hypertensive medication
40	14	Sensitivity analysis of the improvement in risk discrimination

eAppendix 1: Supplementary Methods

Description of studies involved in *de novo* NT-proBNP assays

We conducted NT-proBNP assays in stored serum samples from five cohorts from the DAN-MONICA Study (70yr1914, 45yr1936, GenMon, MONICA II, and MONICA III), the Northern Sweden Health and Disease Study, the Bruneck Study, and the Reykjavik Offspring Study.

The five cohorts from the **DAN-MONICA Study**[1] are case-cohort studies from Denmark nested within population-based prospective cohorts at the Danish Research Centre for Prevention and Health (RCPH). They involved a total of 8314 participants: (i) 70-year olds from the 1914 birth-cohort (70yr1914, n=804); (ii) 45-year olds from the 1936 birth-cohort (45yr1936, n=992); (iii) 5-year re-examination of the first DAN-MONICA cohort (GenMon, n=2987); (iv) the second Dan-MONICA cohort (Monica2, n=1504); and (v) the third Dan-MONICA cohort (Monica3, n=2027). Standardized methods for follow-up and risk factor assessment have been maintained in these five cohorts.[1] The present study included all incident coronary heart disease (CHD) cases recorded as of 31 December 2006, plus subcohorts of randomly sampled participants within each cohort (7% in each of 45yr1936, Monica2, and Monica3; 9% in GenMon; and 28% in 70yr1914; reflecting the proportion of CHD cases within each cohort).

The **Northern Sweden Health and Disease Study**[2-3] comprises participants from two large on-going health monitoring and screening programs in Northern Sweden. The first program entitled "WHO MONICA cohort" comprises three population-based cross-sectional health surveys conducted on a total of 6454 subjects in Norrbotten and Västerbotten counties in 1990, 1994 and 1999 (participation rate >75%). The second program entitled "Västerbotten Intervention Program" recruited 66200 residents of Västerbotten County between 1985 and 1999 to participate in a health screening (participation rate >57%). Incident events of first-ever fatal or non-fatal myocardial infarction were identified through screening of hospital discharge records, general practicioners' records and death certificates, and confirmed using electrocardiography data, levels of cardiac enzymes and symptoms according to standardized MONICA criteria.[4] For each case, one or two healthy controls were selected matching for age (± 2 years), sex, date of health examination (± 4 months) and geographical region. Overall, the study involved 480 incident CHD cases and 869 matched controls.

The **Bruneck Study**[5-6] is a prospective cohort study from Italy. In 1990, 1000 randomly selected inhabitants of Bruneck aged 40-79 years were invited to participate in the study (125 per sex and decade of life), of which 936 people agreed to participate. Follow-up examination took place every five years between 1990 and 2010. NT-proBNP was measured in samples from the 1990 examination in 783 participants free of pre-existing cardiovascular disease. Incidence of cardiovascular outcomes was recorded up to 2010 (follow-up rate 100%). Myocardial infarction was deemed confirmed when World Health Organization criteria for definite disease status were met. Stroke was classified according to the criteria of the National Survey of Stroke. All other revascularization procedures (percutaneous intervention and bypass and surgery) were carefully recorded. Ascertainment of events or procedures did not rely on hospital discharge codes or the patient's self-report, but on a careful review of medical records provided by the general practitioners and files of the Bruneck Hospital and the extensive clinical and laboratory examinations performed as part of the study protocols.

The **Reykjavik Offspring Study**[7] is a prospective cohort study from Iceland that was initiated in 1997 and concluded in 2001. Offspring were selected of individuals who had participated in phases I-V of the Reykjavík study. The Genealogy tracing center found 5351 offspring of 2169 Reykjavík study participants with a history of myocardial infarction, and 2581 offspring of 1170 Reykjavík study participants without a history of myocardial infarction. All offspring were born between 1927 and 1980 and lived in Iceland at that time of selection. Of the 7932 offspring invited, 6025 or 76% entered the study (3964 offspring of cases and 2061 offspring of controls). NT-proBNP was measured in 4358 participants. Those with a baseline history of cardiovascular disease (CVD; defined as history of CHD, stroke, TIA and peripheral vascular disease) were excluded from analysis, leaving 3723 participants included in this study. Follow-up was from the adjudicated MI registry using the WHO criteria to December 31st 2006.

Assay methods used

NT-proBNP was determined using the Elecsys 2010 electrochemiluminescence method (Roche Diagnostics, Burgess Hill, UK).[8]The manufacturer's controls were used to monitor quality control with limits of acceptability defined by the manufacturer. The average low control intra-and inter-assay coefficient of variation (CV) across studies was 3·0% and 4·3% respectively, and high control CV was 2·6% and 2·8% respectively. The lower detection limit of the assay was 5 pg/ml.

Data collation and harmonization

The structure of the data collection and harmonization process is outlined in **Supplementary Appendix Figure 1**

Data transfer: Studies were invited to share individual-participant data on natriuretic peptides, various cardiovascular risk factors, and incident vascular and non-vascular outcomes. Studies also provided information on study design (eg, population source, geographical location), blood storage and handling conditions, natriuretic peptide assays, and methods used to ascertain baseline evidence of CVD. Precise details of the diagnostic criteria used for the definition of cases were sought from each study. Analyses were based on events classified according to codings of International Classification of Diseases or study-specific classification systems. Attribution of death referred to the primary cause provided or, in its absence, the underlying cause provided. Data were accepted in the format they were originally coded and stored by the study investigators. To facilitate the interpretation and processing of the data, studies were asked to provide a data dictionary with information on variable names, measurement units, formats (e.g. DDMMYY in dates), categories (e.g. current, former or never-smoker) as well as missing value codes.

Data cleaning and ratification: The data obtained from each participating study were checked for internal consistency by the coordinating center. Information on categorical variables was systematically recoded to maximize comparability among studies. Any queries were referred back, in confidence, to the study collaborators. Data were converted to a standard format for incorporation into a central database to be used for combined analyses. Detailed summary tabulations based on the converted data were returned to each collaborator for review and confirmation, and any queries regarding the data were solved through correspondence with principal investigators and data managers.

Supplementary Appendix Figure 1: Sequence of data transfer, cleaning and ratification

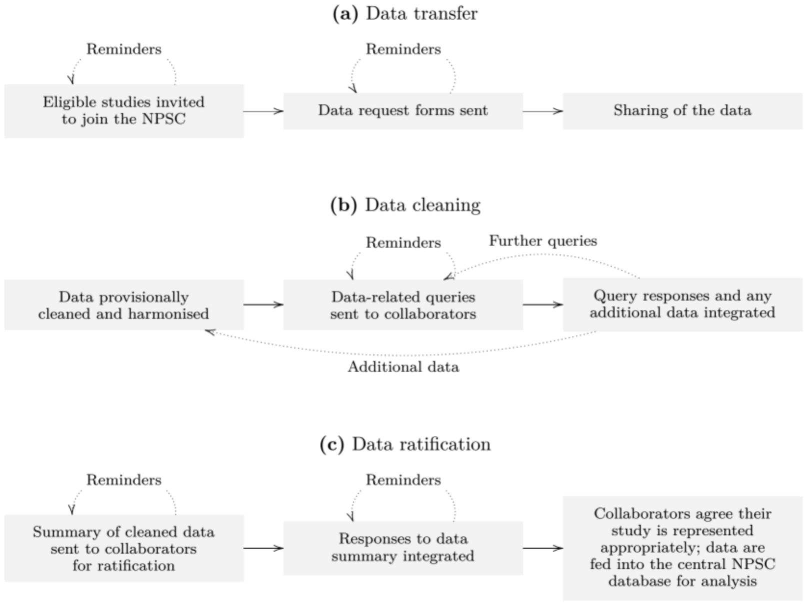

Analytical methods

5.1 Characterizing cross-sectional associations

We quantified sex-specific cross-sectional associations of NT-proBNP with other characteristics as previously described.[10] To avoid over-sampling of incident cases, the analysis was restricted to the random subcohort for case-cohort studies and to controls for nested case-control studies. We used linear mixed models that included random effects at the study level. Continuous variables were divided into tenths based on the overall distribution across all studies. The fixed effects in each model were: study, age, age^2, sex, age x sex, age^2 x sex, risk-factor tenth, risk-factor tenth x sex, and risk-factor tenth x age (where x denotes an interaction). Coefficients that were allowed to vary randomly across cohorts were: age, age^2, sex, and risk-factor tenth (entered as a continuous variable). From each fitted mixed model, overall adjusted geometric mean values and 95% confidence intervals for NT-proBNP concentration by sex within tenths of continuous markers were obtained with age fixed at 60 years. We used these adjusted mean values to assess the shape of the association by plotting geometric mean NT-proBNP against the mean risk factor value within each tenth. Inverse-variance weighted polynomials were superimposed across the adjusted means to help judge whether the overall association was consistent with a linear or quadratic shape. The model to assess the cross-sectional association with age included fixed effects for study, sex, tenths of age, and tenths of age x sex, as well as random effects for tenths of age and sex.

5.2 Association with cardiovascular events

The principal outcome was a first CVD event, defined as a non-fatal myocardial infarction or fatal CHD or any stroke. For each outcome assessed, participants' data were censored if the participant was lost to follow-up, died from other causes, or reached the end of the follow-up period. For participants who had multiple events, analyses focused only on the first CVD event that occurred. Thus, in an analysis of stroke events, participants were followed up until their first stroke event, or were censored at the time of other non-fatal CVD events, such as myocardial infarction or heart failure (in addition to standard censoring at death from other causes or loss to follow-up). The rationale for this was that any major CVD event may lead to lifestyle and other modifications (eg, medication use) that may alter levels of factors significantly and disrupt the association between baseline risk factors and subsequent disease risk. Individuals were not censored at the time of cardiovascular investigations or interventions, such as angiography or coronary bypass operations, or at the diagnosis of angina, because such information was considered not to be recorded reliably enough in sufficient studies.

Analyses used a two-stage approach with estimates of association calculated separately within each study before pooling them across studies by multivariate random-effects meta-analysis.[11-12] For studies analysed as prospective cohort studies, hazard ratios were calculated with Cox proportional hazard regression models stratified by sex, using time-on-study as time scale. For case-cohort studies, Cox proportional hazard regression models with Prentice weights and robust standard errors were used.[13] The proportional hazards assumption was satisfied in each of the studies.[11] For nested case-control studies, odds ratios were calculated using conditional or unconditional logistic regression models, as appropriate. Hazard ratios and odds ratios were assumed to represent the same relative risk and were collectively described as risk ratios. To avoid over-fitting of the statistical models, we excluded studies with fewer than 10 incident events of an outcome from the analysis of that particular outcome (although such studies were able to contribute full data for analyses of cross-sectional correlates). Risk ratios were calculated for overall thirds of NT-proBNP values. We conducted several sensitivity analyses, including analyses that: defined thirds separately for males and females; used 1-SD higher log NT-proBNP; excluded people with high baseline levels of NT-proBNP[14-15] (ie, >450 pg/ml for people aged 50 years or younger, >900 pg/ml for people aged 50-75 years, and >1800 pg/ml for people aged 75 years or older); excluded the initial 5 years of follow-up; and restricted attention to studies recording both fatal and non-fatal outcomes. To test for a difference in associations of NT-proBNP with fatal vs. non-fatal CHD, we defined a competing risk model using record duplication approach, allowing for simultaneous cause-specific hazard regression to estimate hazard ratios for each type of event and also testing for differences based on interaction between NT-pro BNP dummy variables and event type indicator variable.

Shape of association

To characterize shapes of associations, study-specific risk ratios were calculated by overall tenths of NT-proBNP, pooled on the log scale by multivariate random-effects meta-analysis, and plotted against the geometric mean of NT-proBNP values within each category.[11] The size of boxes in the figure is proportional to the inverse of the variance of the respective estimate. 95% confidence intervals were estimated from "floated" variances that assign appropriate confidence intervals to the log risk ratio in every group, including the reference group, and enable valid comparisons to be made between any two exposure groups.[16]

Between-study heterogeneity and effect modification

Between-study heterogeneity was assessed by the I^2 statistic, which quantifies the percentage of variance in the point estimates of the study-specific effect estimates that is attributable to between-study variation as opposed to sampling variation (with values close to 0 indicating lack of evidence of heterogeneity).[17-18]

Effect modification by individual characteristics was investigated by formal tests of interaction. P values for interaction were calculated using continuous variables, when appropriate. Only studies that had different levels of the potential effect modifier were included in this analysis. Diversity at the study level (such as differences by study design or laboratory methods) was investigated by grouping studies by recorded characteristics and by meta-regression.[19] Ethnicity was treated as a study-level characteristic, because ethnic diversity within included studies was low.

Incremental value in risk prediction

To quantify the added value of NT-proBNP measurement for CVD prediction, we calculated measures of discrimination and risk reclassification using only cohort and case-cohort studies (ie, the two nested case-control studies were excluded from these analyses).[20-21] In the primary analysis, the base model included information on the conventional risk factors variables age, smoking, systolic blood pressure, history of diabetes, and levels of total and HDL-cholesterol, and was stratified by study and sex. NT-proBNP concentration was log-transformed and modeled using both linear and quadratic terms. We used a 2-stage approach that allowed for the examination of between-study heterogeneity through calculation of the C-index, a measure of risk discrimination, and changes therein within each study before pooling results weighted by the numbers of CVD outcomes contributed.[22-23] χ^2 tests were used to test for differences in changes in discrimination measures across subgroups.

We calculated measures of risk reclassification (i.e. integrated discrimination improvement and categorial and continuous net reclassification improvement)[23] in studies in which both fatal and nonfatal cardiovascular events had been recorded, using a 1-stage approach.[22-23] For the calculation of the net reclassification improvement, we used predicted 10-year CVD risk categories as defined by the following clinical guidelines: <5%, 5% to <7·5%, and ≥7·5% according to the 2013 ACC/AHA guideline;[24] <5%, 5% to <10%, and ≥10% according to the 2014 NICE guideline;[25] <10%, 10% to <20%, and ≥20% according to the 2010 ACCF/AHA guideline;[26] and <2·5%, 2·5% to <5%, and ≥5% according to the 2016 ESC guideline.[27]

eAppendix 2: Systematic literature search procedures

Studies eligible for inclusion in the Natriuretic Peptides Studies Collaboration (NPSC) were identified by systematic searches of PubMed, Scientific Citation Index Expanded and EMBASE. The literature search was conducted up to September 4th 2014, did not apply language restrictions, and combined terms related to natriuretic peptide family members and the primary outcomes of interest (**Supplementary Appendix Figure 2**). In addition, the reference lists of identified articles (including review articles and the previously published meta-analysis)[9] were scanned for additional relevant studies. Prospective cohort studies were eligible if they met the following criteria: (i) had assayed either NT-proBNP or BNP; (ii) recorded baseline information on conventional risk factors (ie, age, sex, smoking status, blood pressure, history of diabetes, total and HDL-cholesterol); (iii) included participants without a recorded baseline history of CVD; and (iv) recorded cause-specific deaths or cardiovascular events (nonfatal myocardial infarction or stroke) or both during more than 1 year of follow-up using well-defined criteria.

After removal of any duplicates, 24864 articles were assessed for eligibility (**Supplementary Appendix Figure 3**) Of these, 24696 were excluded because they did not fulfill the pre-specified inclusion criteria. A total of 33 non-overlapping cohorts (published in 168 articles) were invited to the NPSC, of which 32 joined the collaboration. Together with the *de novo* data from 8 additional cohorts, data from 40 cohorts were available for analysis.

Supplementary Appendix Figure 2: Search strategy to identify studies eligible for inclusion in the NPSC

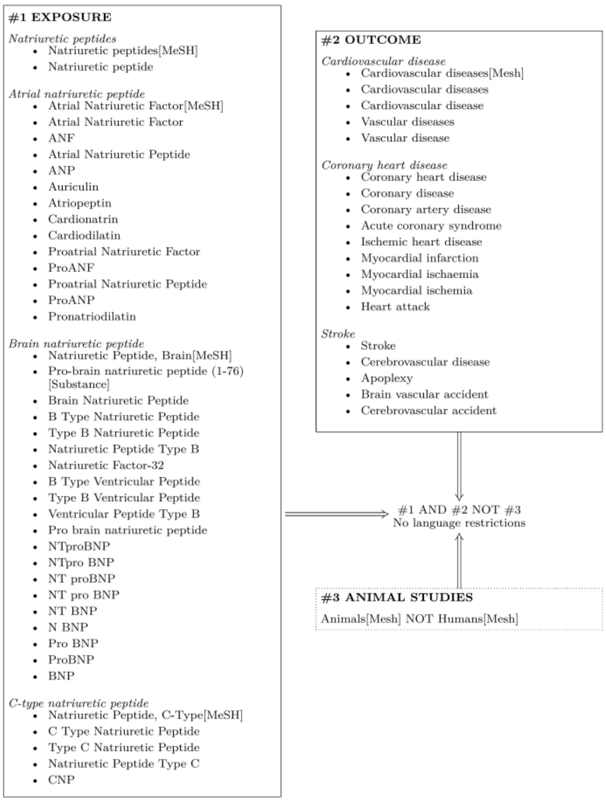

Supplementary Appendix Figure 3: Flow diagram from study identification to data sharing

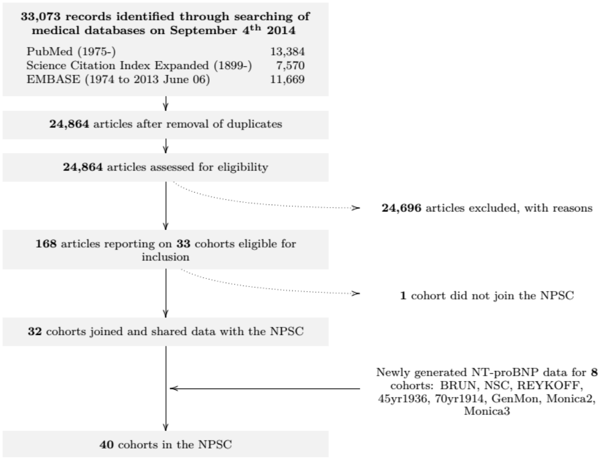

eAppendix 3: IPD Flow Diagram

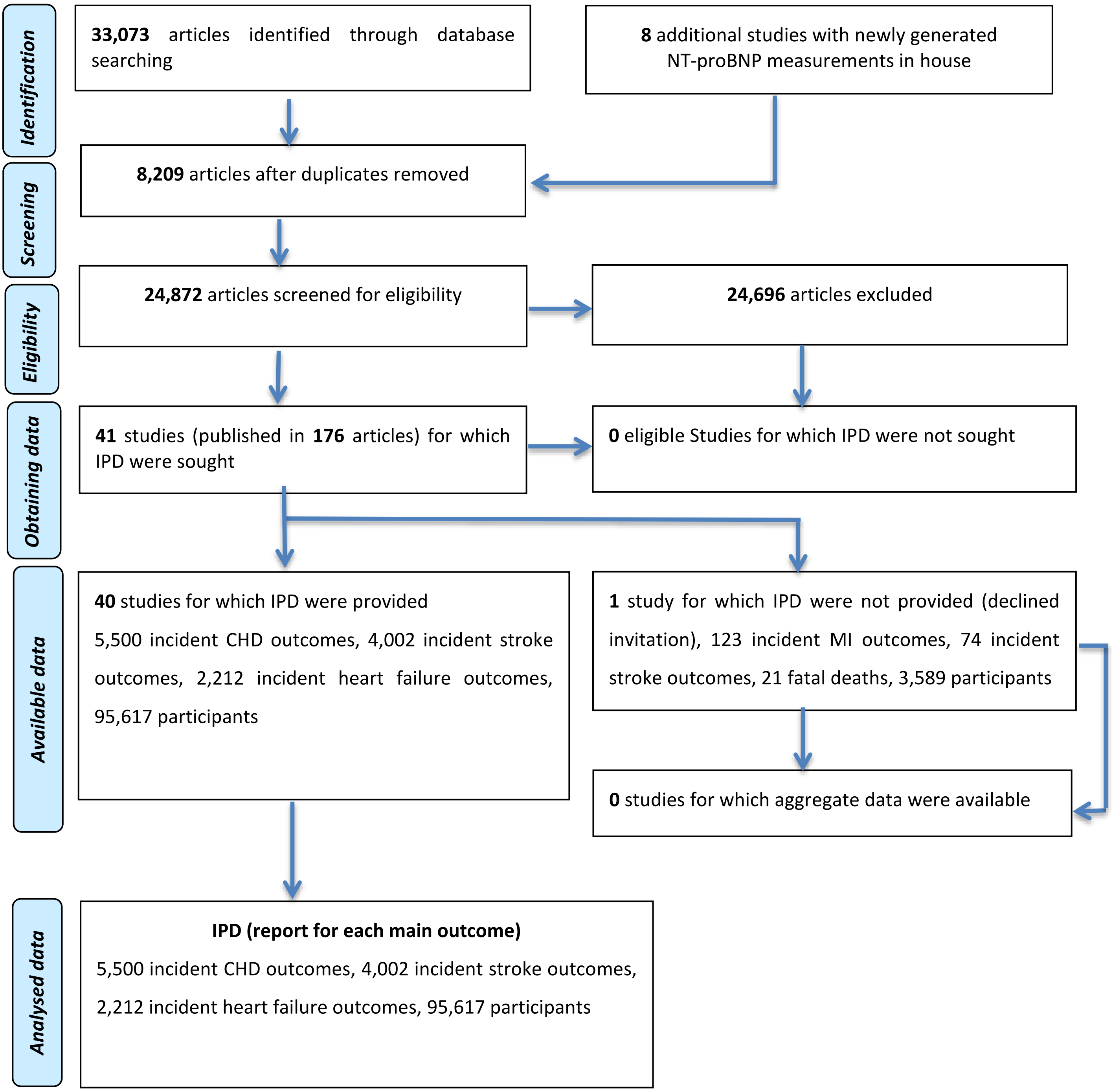

eAppendix 4: PRISMA-IPD checklist

PRISMA-IPD Section/Topic	Checklist item	Reported on page
**Title**		
Title	Identify the report as a systematic review and meta-analysis of individual participant data.	1
**Abstract**		
Structured summary	Provide a structured summary including as applicable:	
	Background: state research question and main objectives, with information on participants, interventions, comparators, and outcomes.	1
	Methods: report eligibility criteria; data sources including dates of last bibliographic search or elicitation, noting that IPD were sought; methods of assessing risk of bias.	1
	Results: provide number and type of studies and participants identified and number (%) obtained; summary effect estimates for main outcomes (benefits and harms) with confidence intervals and measures of statistical heterogeneity. Describe the direction and size of summary effects in terms meaningful to those who would put findings into practice.	1
	Discussion: state main strengths and limitations of the evidence, general interpretation of the results, and any important implications.	1
**Introduction**		
Rationale	Describe the rationale for the review in the context of what is already known.	1
Objectives	Provide an explicit statement of the questions being addressed with reference, as applicable, to participants, interventions, comparisons, outcomes, and study design (PICOS). Include any hypotheses that relate to particular types of participant-level subgroups.	1
**Methods**		
Protocol and registration	Indicate if a protocol exists and where it can be accessed. If available, provide registration information including registration number and registry name. Provide publication details, if applicable.	NA
Eligibility criteria	Specify inclusion and exclusion criteria including those relating to participants, interventions, comparisons, outcomes, study design, and characteristics (eg, years when conducted, required minimum follow-up). Note whether these were applied at the study or individual level, ie, whether eligible participants were included (and ineligible participants excluded) from a study that included a wider population than specified by the review inclusion criteria. The rationale for criteria should be stated.	2
Identifying studies: information sources	Describe all methods of identifying published and unpublished studies including, as applicable: which bibliographic databases were searched with dates of coverage; details of any hand searching including of conference proceedings; use of study registers and agency or company databases; contact with the original research team and experts in the field; open advertisements; and surveys. Give the date of last search or elicitation.	eAppendix 2
Identifying studies: search	Present the full electronic search strategy for at least 1 database, including any limits used, such that it could be repeated.	eAppendix 2
Study selection processes	State the process for determining which studies were eligible for inclusion.	2, eAppendix 2
Data collection processes	Describe how IPD were requested, collected, and managed, including any processes for querying and confirming data with investigators. If IPD were not sought from any eligible study, the reason for this should be stated (for each such study).	eAppendix 2
	If applicable, describe how any studies for which IPD were not available were dealt with. This should include whether, how, and what aggregate data were sought or extracted from study reports and publications (such as extracting data independently in duplicate) and any processes for obtaining and confirming these data with investigators.	eAppendix 2, eAppendix 3

Data items	Describe how the information and variables to be collected were chosen. List and define all study-level and participant-level data that were sought, including baseline and follow-up information. If applicable, describe methods of standardizing or translating variables within the IPD data sets to ensure common scales or measurements across studies.	eAppendix 2
IPD integrity	Describe what aspects of IPD were subject to data checking (such as sequence generation, data consistency and completeness, baseline imbalance) and how this was done.	eAppendix 2
Risk of bias assessment in individual studies	Describe methods used to assess risk of bias in the individual studies and whether this was applied separately for each outcome. If applicable, describe how findings of IPD checking were used to inform the assessment. Report if and how risk of bias assessment was used in any data synthesis.	eAppendix 1
Specification of outcomes and effect measures	State all treatment comparisons of interest. State all outcomes addressed and define them in detail. State whether they were prespecified for the review and, if applicable, whether they were primary/main or secondary/additional outcomes. Give the principal measures of effect (such as risk ratio, hazard ratio, difference in means) used for each outcome.	3
Synthesis methods	Describe the meta-analysis methods used to synthesize IPD. Specify any statistical methods and models used.	3
Exploration of variation in effects	If applicable, describe any methods used to explore variation in effects by study- or participant-level characteristics (such as estimation of interactions between effect and covariates). State all participant-level characteristics that were analyzed as potential effect modifiers and whether these were prespecified.	3
Risk of bias across studies	Specify any assessment of risk of bias relating to the accumulated body of evidence, including any pertaining to not obtaining IPD for particular studies, outcomes, or other variables.	3
Additional analyses	Describe methods of any additional analyses, including sensitivity analyses. State which of these were prespecified.	3 and eAppendix 1
**Results**		
Study selection and IPD obtained	Give numbers of studies screened, assessed for eligibility, and included in the systematic review with reasons for exclusions at each stage. Indicate the number of studies and participants for which IPD were sought and for which IPD were obtained. For those studies for which IPD were not available, give the numbers of studies and participants for which aggregate data were available. Report reasons for nonavailability of IPD. Include a flow diagram.	eAppendix 2 and eAppendix 3
Study characteristics	For each study, present information on key study and participant characteristics (such as description of interventions, numbers of participants, demographic data, unavailability of outcomes, funding source, and if applicable duration of follow-up). Provide (main) citations for each study. Where applicable, also report similar study characteristics for any studies not providing IPD.	eTable 1-4
IPD integrity	Report any important issues identified in checking IPD or state that there were none.	eAppendix 2
Risk of bias within studies	Present data on risk of bias assessments. If applicable, describe whether data checking led to the up-weighting or down-weighting of these assessments. Consider how any potential bias affects the robustness of meta-analysis conclusions.	eTable 8, eFigures 5, 7, 12 & 14
Results of individual studies	For each comparison and for each main outcome (benefit or harm), for each individual study report the number of eligible participants for which data were obtained and show simple summary data for each intervention group (including, where applicable, the number of events), effect estimates, and confidence intervals. These may be tabulated or included on a forest plot.	eFigures 4, 10 & 11
Results of syntheses	Present summary effects for each meta-analysis undertaken, including confidence intervals and measures of statistical heterogeneity. State whether the analysis was prespecified, report the numbers of studies and participants and, where applicable, report the number of events on which it is based.	Table 1, Figures 1-4, and Supplement ary material
	When exploring variation in effects due to patient or study characteristics, present summary interaction estimates for each characteristic examined, including confidence intervals and measures of statistical heterogeneity. State whether the analysis was prespecified. State whether any interaction	Supplement ary material

	is consistent across trials.	
	Provide a description of the direction and size of effect in terms meaningful to those who would put findings into practice.	Table 1, Figures 1-4, and Supplementary material
Risk of bias across studies	Present results of any assessment of risk of bias relating to the accumulated body of evidence, including any pertaining to the availability and representativeness of available studies, outcomes, or other variables.	Supplementary material
Additional analyses	Additional analyses	Supplementary material
**Discussion**		
Summary of evidence	Summarize the main findings, including the strength of evidence for each main outcome.	7
Strengths and limitations	Discuss any important strengths and limitations of the evidence, including the benefits of access to IPD and any limitations arising from IPD that were not available.	7-8
Conclusions	Provide a general interpretation of the findings in the context of other evidence.	7-8
Implications	Consider relevance to key groups (such as policy makers, service providers, and service users). Consider implications for future research.	7-8
**Funding**		
Funding	Describe sources of funding and other support (such as supply of IPD) and the role in the systematic review of those providing such support.	1, 3

eAppendix 5: List of contributing studies

Study/cohort	Reference	Study group full name
ARIC	30	Atherosclerosis Risk in Communities Study
ATTICA	31	ATTICA Study
BRHS	32	British Regional Heart Study
BRUN	5-6	Bruneck Study
BWHHS	33	British Women's Heart and Health Study
CHS	34	Cardiovascular Health Study
CHS1	*34*	*Cardiovascular Health Study (Original cohort)*
CHS2	*34*	*Cardiovascular Health Study (Supplemental African-American cohort)*
COPEN	35	Copenhagen City Heart Study
DANMON	1	Danish MONICA Study / MONICA10
45yr1936	*1*	*45 year old from the 1936 birth-cohort*
70yr1914	*1*	*70 year old from the 1914 birth-cohort*
GenMon	*1*	*Five year re-examination of the first MONICA cohort*
Monica2	*1*	*The second MONICA cohort*
Monica3	*1*	*The third MONICA cohort*
DHS	36	Dallas Heart Study
FINRISK97	37	Finrisk Cohort 1997
FRAMOFF	38	Framingham Offspring Cohort
HISAYAMA	39	Hisayama Study (Survey 2002)
KIHD	28	Kuopio Ischaemic Heart Disease Risk Factor Study
KISTORP	40	Study by Kistorp et al.
LIFE	41	Losartan Intervention For Endpoint Reduction in Hypertension Study
MDCS	42	Malmö Diet and Cancer Study Cardiovascular Cohort
MESA	43	Multi-Ethnic Study of Atherosclerosis
MONICA/KORA3	44	MONICA/KORA Augsburg Survey S3
NSHDS	3	Northern Sweden Health and Disease Study
OHS	45	Olivetti Heart Study
PIVUS	46	Prospective Investigation of the Vasculature in Uppsala Seniors
PREVEND	47	Prevention of Renal and Vascular End Stage Disease
PRIME	37	Prospective Epidemiological Study of Myocardial Infarction
PROSPER	48	Prospective Study of Pravastatin in the Elderly at Risk
PTLBNP	49	Prognostic Threshold Levels of NT-proBNP testing in primary care
RANCHO	50	Rancho Bernardo Study
REYKOFF	7	Reykjavik Offspring Study
REGARDS	51	Reasons for Geographic and Racial Differences in Stroke Study
RS-I	52	Rotterdam Study I
RS-II	52	Rotterdam Study II
SHS	53	Strong Heart Study (North and South Dakota Centres)
ULSAM	54	Uppsala Longitudinal Study of Adult Men
WHIOS	55	Women's Health Initiative Observational Study
WHS	56	Women's Health Study
WOSCOPS	57	West of Scotland Coronary Prevention Study

eAppendix 6: Supplementary references

1. Osler M, Linneberg A, Glümer C, Jørgensen T. The cohorts at the Research Centre for Prevention and Health, formerly 'The Glostrup Population Studies'. *Int J Epidemiol* 2011; **40**: 602–10.
2. Wennberg P, Wensley F, Di Angelantonio E, et al. Haemostatic and inflammatory markers are independently associated with myocardial infarction in men and women. *Thromb Res* 2012; **129**: 68–73.
3. Hallmans G, Agren A, Johansson G, et al. Cardiovascular disease and diabetes in the Northern Sweden Health and Disease Study Cohort - evaluation of risk factors and their interactions. *Scand J Public Health Suppl* 2003; **61**: 18–24.
4. Tunstall-Pedoe H, Kuulasmaa K, Amouyel P, Arveiler D, Rajakangas AM, Pajak A. Myocardial infarction and coronary deaths in the World Health Organization MONICA Project. Registration procedures, event rates, and case-fatality rates in 38 populations from 21 countries in four continents. *Circulation* 1994; **90**: 583–612.
5. Willeit P, Kiechl S, Kronenberg F, et al. Discrimination and net reclassification of cardiovascular risk with lipoprotein(a): prospective 15-year outcomes in the Bruneck Study. *J Am Coll Cardiol* 2014; **64**: 851–60.
6. Kiechl S, Willeit J. The natural course of atherosclerosis. Part I: incidence and progression. *Arterioscler Thromb Vasc Biol* 1999; **19**: 1484–90.
7. Jónsdóttir LS, Sigfússon N, Gudnason V, Sigvaldason H, Thorgeirsson G. Do lipids, blood pressure, diabetes, and smoking confer equal risk of myocardial infarction in women as in men? The Reykjavik Study. *J Cardiovasc Risk* 2002; **9**: 67–76.
8. Roche Diagnostics. proBNP II STAT Product Insert 2011.
9. Di Angelantonio E, Chowdhury R, Sarwar N, Aspelund T, Danesh J, Gudnason V. Chronic kidney disease and risk of major cardiovascular disease and non-vascular mortality: prospective population based cohort study. *BMJ* 2010; **341**: c4986.
10. Fibrinogen Studies Collaboration, Kaptoge S, White IR, et al. Associations of plasma fibrinogen levels with established cardiovascular disease risk factors, inflammatory markers, and other characteristics: individual participant meta-analysis of 154,211 adults in 31 prospective studies: the fibrinogen studies collaboration. *Am J Epidemiol* 2007; **166**: 867–79.
11. Thompson S, Kaptoge S, White I, et al. Statistical methods for the time-to-event analysis of individual participant data from multiple epidemiological studies. *Int J Epidemiol* 2010; **39**: 1345–59.
12. White IR. Multivariate random-effects meta-analysis. *Stata Journal* 2009; **9**: 40–56.
13. Prentice R. A case-cohort design for epidemiologic cohort studies and disease prevention trials. *Biometrika* 1986; **73**: 1–11.
14. Yancy CW, Jessup M, Bozkurt B, et al. 2013 ACCF/AHA guideline for the management of heart failure: a report of the American College of Cardiology Foundation/American Heart Association Task Force on Practice Guidelines. *J Am Coll Cardiol* 2013; **62**: e147–e239.
15. Januzzi JL, van Kimmenade R, Lainchbury J, et al. NT-proBNP testing for diagnosis and short-term prognosis in acute destabilized heart failure: an international pooled analysis of 1256 patients: the International Collaborative of NT-proBNP Study. *Eur Heart J* 2006; **27**: 330–7.
16. Easton DF, Peto J, Babiker AG. Floating absolute risk: an alternative to relative risk in survival and case-control analysis avoiding an arbitrary reference group. *Stat Med* 1991; **10**: 1025–35.

17. Higgins JPT, Thompson SG, Deeks JJ, Altman DG. Measuring inconsistency in meta-analyses. *BMJ* 2003; **327**: 557–60.
18. Higgins JPT, Thompson SG. Quantifying heterogeneity in a meta-analysis. *Stat Med* 2002; **21**: 1539–58.
19. Thompson SG, Sharp SJ. Explaining heterogeneity in meta-analysis: a comparison of methods. *Stat Med* 1999; **18**: 2693–708.
20. Pennells L, White IR, Wood AM, et al. Measures to assess the prognostic ability of the stratified Cox proportional hazards model. *Stat Med* 2009; **28**: 389–411.
21. Sanderson J, Thompson SG, White IR, Aspelund T, Pennells L. Derivation and assessment of risk prediction models using case-cohort data. *BMC Med Res Methodol* 2013; **13**: 113.
22. Pennells L, Kaptoge S, White IR, Thompson SG, Wood AM. Assessing risk prediction models using individual participant data from multiple studies. *Am J Epidemiol* 2014; **179**: 621–32.
23. Pencina MJ, D'Agostino RB Sr, Steyerberg EW. Extensions of net reclassification improvement calculations to measure usefulness of new biomarkers. *Stat Med* 2011; **30**: 11–21.
24. Goff DC Jr, Lloyd-Jones DM, Bennett G, et al. 2013 ACC/AHA Guideline on the Assessment of Cardiovascular Risk. *J Am Coll Cardiol* 2014; **63**: 2935–59.
25. National Institute for Health and Care Excellence. Cardiovascular disease: risk assessment and reduction, including lipid modification. London: National Institute for Health and Care Excellence, 2014.
26. Greenland P, Alpert JS, Beller GA, et al. 2010 ACCF/AHA guideline for assessment of cardiovascular risk in asymptomatic adults: a report of the American College of Cardiology Foundation/American Heart Association Task Force on Practice Guidelines. *Circulation* 2010; **122**: e584–e636.
27. Piepoli MF, Hoes AW, Agewall S, et al. 2016 European Guidelines on cardiovascular disease prevention in clinical practice. *Eur Heart J* 2016; 37: 2315–81.
28. Laukkanen JA, Kurl S, Ala-Kopsala M, et al. Plasma N-terminal fragments of natriuretic propeptides predict the risk of cardiovascular events and mortality in middle-aged men. *Eur Heart J* 2006; **27**: 1230–7.
29. Mueller T, Gegenhuber A, Poelz W, Haltmayer M. Comparison of the Biomedica NT-proBNP enzyme immunoassay and the Roche NT-proBNP chemiluminescence immunoassay: implications for the prediction of symptomatic and asymptomatic structural heart disease. *Clin Chem* 2003; **49**: 976–9.
30. Saunders JT, Nambi V, de Lemos JA, et al. Cardiac troponin T measured by a highly sensitive assay predicts coronary heart disease, heart failure, and mortality in the Atherosclerosis Risk in Communities Study. *Circulation* 2011; **123**: 1367–76.
31. Fragopoulou E, Panagiotakos DB, Pitsavos C, et al. N-terminal ProBNP distribution and correlations with biological characteristics in apparently healthy Greek population: ATTICA study. *Angiology* 2010; **61**: 397–404.
32. Wannamethee SG, Welsh P, Lowe GD, et al. N-terminal pro-brain natriuretic Peptide is a more useful predictor of cardiovascular disease risk than C-reactive protein in older men with and without pre-existing cardiovascular disease. *J Am Coll Cardiol* 2011; **58**: 56–64.
33. Sattar N, Welsh P, Sarwar N, et al. NT-proBNP is associated with coronary heart disease risk in healthy older women but fails to enhance prediction beyond established risk factors: results from the British Women's Heart and Health Study. *Atherosclerosis* 2010; **209**: 295–9.
34. Patton KK, Sotoodehnia N, DeFilippi C, Siscovick DS, Gottdiener JS, Kronmal RA. N-terminal pro-B-type natriuretic peptide is associated with sudden cardiac death risk: the Cardiovascular Health Study. *Heart Rhythm* 2011; **8**: 228–33.

35. Mogelvang R, Goetze JP, Schnohr P, et al. Discriminating between cardiac and pulmonary dysfunction in the general population with dyspnea by plasma pro-B-type natriuretic peptide. *J Am Coll Cardiol* 2007; **50**: 1694–701.
36. de Lemos JA, Drazner MH, Omland T, et al. Association of troponin T detected with a highly sensitive assay and cardiac structure and mortality risk in the general population. *JAMA* 2010; **304**: 2503–12.
37. Blankenberg S, Zeller T, Saarela O, et al. Contribution of 30 biomarkers to 10-year cardiovascular risk estimation in 2 population cohorts: the MONICA, risk, genetics, archiving, and monograph (MORGAM) biomarker project. *Circulation* 2010; **121**: 2388–97.
38. Wang TJ, Larson MG, Levy D, et al. Plasma natriuretic peptide levels and the risk of cardiovascular events and death. *N Engl J Med* 2004; **350**: 655–63.
39. Doi Y, Ninomiya T, Hata J, et al. N-Terminal Pro-Brain Natriuretic Peptide and Risk of Cardiovascular Events in a Japanese Community: The Hisayama Study. *Arterioscler Thromb Vasc Biol* 2011; **31**: 2997–3003.
40. Kistorp C, Raymond I, Pedersen F, Gustafsson F, Faber J, Hildebrandt P. N-terminal pro-brain natriuretic peptide, C-reactive protein, and urinary albumin levels as predictors of mortality and cardiovascular events in older adults. *JAMA* 2005; **293**: 1609–1616.
41. Olsen MH, Wachtell K, Nielsen OW, et al. N-terminal brain natriuretic peptide predicted cardiovascular events stronger than high-sensitivity C-reactive protein in hypertension: a LIFE substudy. *J Hypertens* 2006; **24**: 1531–1539.
42. Melander O, Newton-Cheh C, Almgren P, et al. Novel and conventional biomarkers for prediction of incident cardiovascular events in the community. *JAMA* 2009; **302**: 49–57.
43. Choi EY, Bahrami H, Wu CO, et al. N-terminal pro-B-type natriuretic peptide, left ventricular mass, and incident heart failure: Multi-Ethnic Study of Atherosclerosis. *Circ Heart Fail* 2012; **5**: 727–34.
44. Luchner A, Behrens G, Stritzke J, et al. Long-term pattern of brain natriuretic peptide and N-terminal pro brain natriuretic peptide and its determinants in the general population: contribution of age, gender, and cardiac and extra-cardiac factors. *Eur J Heart Fail* 2013; **15**: 859–67.
45. Barbato A, Sciarretta S, Marchitti S, et al. Aminoterminal natriuretic peptides and cardiovascular risk in an Italian male adult cohort. *Int J Cardiol* 2011; **152**: 245–6.
46. Eggers KM, Lindahl B, Venge P, Lind L. B-type natriuretic peptides and their relation to cardiovascular structure and function in a population-based sample of subjects aged 70 years. *Am J Cardiol* 2009; **103**: 1032–8.
47. Linssen GCM, Bakker SJL, Voors AA, et al. N-terminal pro-B-type natriuretic peptide is an independent predictor of cardiovascular morbidity and mortality in the general population. *Eur Heart J* 2010; **31**: 120–7.
48. Poortvliet RK, van Peet PG, de Craen AJ, et al. Risk stratification and treatment effect of statins in secondary cardiovascular prevention in old age: Additive value of N-terminal pro-B-type natriuretic peptide. *Eur J Prev Cardiol* 2016; **34**: 1104–13.
49. Rosenberg J, Schou M, Gustafsson F, Badskjaer J, Hildebrandt P. Prognostic threshold levels of NT-proBNP testing in primary care. *Eur Heart J* 2009; **30**: 66–73.
50. Daniels LB, Laughlin GA, Clopton P, Maisel AS, Barrett-Connor E. Minimally elevated cardiac troponin T and elevated N-terminal pro-B-type natriuretic peptide predict mortality in older adults: results from the Rancho Bernardo Study. *J Am Coll Cardiol* 2008; **52**: 450–9.
51. Howard VJ, Cushman M, Pulley L, et al. The reasons for geographic and racial differences in stroke study: objectives and design. *Neuroepidemiology* 2005; **25**: 135–3.
52. Rutten JHW, Mattace-Raso FUS, Steyerberg EW, et al. Amino-terminal pro-B-type natriuretic peptide improves cardiovascular and cerebrovascular risk prediction in the population: the Rotterdam study. *Hypertension* 2010; **55**: 785–91.

53. Kizer JR, Krauser DG, Rodeheffer RJ, et al. Prognostic value of multiple biomarkers in American Indians free of clinically overt cardiovascular disease (from the Strong Heart Study). *Am J Cardiol* 2009; **104**: 247–53.

54. Zethelius B, Berglund L, Sundström J, et al. Use of multiple biomarkers to improve the prediction of death from cardiovascular causes. *N Engl J Med* 2008; **358**: 2107–16.

55. Everett BM, Berger JS, Manson JE, Ridker PM, Cook NR. B-type natriuretic peptides improve cardiovascular disease risk prediction in a cohort of women. *J Am Coll Cardiol* 2014; **64**: 1789–97.

56. Everett BM, Ridker PM, Cook NR, Pradhan AD. Usefulness of B-type Natriuretic Peptides to Predict Cardiovascular Events in Women (from the Women's Health Study). *Am J Cardiol* 2015; **116**: 532–7.

57. Welsh P, Doolin O, Willeit P, et al. N-terminal pro-B-type natriuretic peptide and the prediction of primary cardiovascular events: results from 15-year follow-up of WOSCOPS. *Eur Heart J* 2013; **34**: 443–50.

eTable 1. Study design and natriuretic peptide measurement in the 40 cohorts contributing to the NPSC.

Study design / study acronym	Country	Year(s) of baseline survey	Population source	Fasting status	Sample type	Storage duration (years)	Storage temperature (˚C)	Manufacturer of NTproBNP assay	Manufacturer of BNP assay
**Prospective cohort study**									
ARIC	USA	1996-1998	Households	8hrs+	Plasma	11–14	-80°	RocheDiag (Generation II)	–
ATTICA	Greece	2001-2002	Households	8hrs+	Serum	5	-80°	RocheDiag (Generation I)	–
BRHS	UK	1998-2000	GP/Health service lists	4-8hrs	Serum	9–11	-70°	RocheDiag (Generation II)	–
BRUN	Italy	1990	Population register	8hrs+	Serum	24	-70°	RocheDiag (Generation II)	–
CHS	USA	1989-1993	GP/Health service lists	8hrs+	Serum	12–19	-70°	RocheDiag (Generation I)	–
COPEN	Denmark	2001-2003	Population register	Non-fasted/random	Plasma	4	-70°	In house*	–
DHS	USA	2000-2002	Population register	Fasted/NR	Plasma	1–3	-70°	RocheDiag (Generation I)	Biosite
FINRISK97	Finland	1997	Population register	Non-fasted/random	Serum/Plasma	11	-70°	RocheDiag (Generation II)	Abbott
FRAMOFF	USA	1995-1998	Population register	Non-fasted/random	Plasma	1–4	-70°	–	Shionogi
HISAYAMA	Japan	2002	Population register	Non-fasted/random	Serum	7	-80°	RocheDiag (Generation II)	–
KIHD	Finland	1991-1993	Population register	8hrs+	Plasma	10–12	-20°	In house†	–
KISTORP	Denmark	1998-2000	GP/Health service lists	Non-fasted/random	Plasma	3	-80°	RocheDiag (Generation I)	Shionogi
LIFE	Scandinavia	1995-1997	GP/Health service lists	Non-fasted/random	Serum	7–8	-20°	RocheDiag (Generation I)	–
MDCS	Sweden	1991-1994	Population register	8hrs+	Plasma	Fresh	-80°	DadeBehring	–
MESA	USA	2000-2002	Population register	8hrs+	Serum	10	-70°	RocheDiag (Generation II)	–
MONICA/KORA3	Germany	1994-1995	Population register	Non-fasted/random	Plasma	7	-70°	RocheDiag (Generation I)	Shionogi
OHS	Italy	2002-2004	Occupational	8hrs+	Serum	5–6	-80°	Biomedica‡	–
PIVUS	Sweden	2001-2005	Population register	8hrs+	Plasma	1–4	-80°	RocheDiag (Generation I)	Abbott
PREVEND	Netherlands	1997-1998	Population register	8hrs+	Plasma	6	-80°	RocheDiag (Generation I)	–
PRIME	UK	1991-1994	Population register	8hrs+	Serum	14–17	-70°	RocheDiag (Generation II)	Shionogi
PROSPER	UK/IRL/NLD	1998	Screening programme	8hrs+	Plasma	15-16	-80°	RocheDiag (Generation II)	–
PTLBNP	Denmark	2003-2005	GP/Health service lists	Non-fasted/random	Plasma	Fresh	NA	RocheDiag (Generation II)	–
RANCHO	USA	1997-1999	Households	8hrs+	Plasma	7–9	-70°	RocheDiag (Generation I)	–
REYKOFF	Iceland	1997-2003	Population register	8hrs+	Serum	8–12	-70°	RocheDiag (Generation II)	–
RS-I	Netherlands	1997-1999	Population register	4-8hrs	Serum	10	-80°	RocheDiag (Generation II)	–
RS-II	Netherlands	2000-2001	Population register	4-8hrs	Serum	7	-80°	RocheDiag (Generation II)	–
SHS	USA	1993-1995	Population register	8hrs+	Plasma	10	-80°	–	Shionogi
ULSAM	Sweden	1991-1995	Population register	8hrs+	Plasma	11	-70°	RocheDiag (Generation I)	–
WOSCOPS	UK	1989-1995	GP/Health service lists	8hrs+	Serum	18–20	-70°	RocheDiag (Generation II)	–
**Case-cohort study**									
DANMON	Denmark	1981-1992	Population register	8hrs+	Plasma	17–28	-70°	RocheDiag (Generation II)	–
REGARDS	USA	2003-2007	Households	8hrs+	Serum	5-9	-80°	RocheDiag (Generation II)	–
WHIOS	USA	1994-1998	Population register	8hrs+	Plasma	12-16	-70°	RocheDiag (Generation II)	–
WHS	USA	1992-1995	Occupational	Non-fasted/random	Plasma	14-17	-70°	RocheDiag (Generation II)	–
**Nested case-control**									
BWHHS	UK	1999-2001	Population register	4-8hrs	Serum	9-11	-80°	RocheDiag (Generation II)	–
NSHDS	Sweden	1985-1999	Population register	4-8hrs	Plasma	7-24	-20°	RocheDiag (Generation II)	–

Full study names are listed in the **eAppendix 5**. CHS consists of the cohorts CHS1 and CHS2. DANMON consists of the cohorts 45yr1936, 70yr1914, GenMon, Monica2, and Monica3. Abbreviations: GP, general practitioner; NA, not applicable; NR, not reported. *COPEN used an in-house assay capturing both proBNP and NT-proBNP. †KIHD measured NT-proBNP with a radioimmunoassay based on an antiserum directed to NT-proBNP$_{10–29}$. ‡To achieve comparability between Biomedica and Roche assays, NT-proBNP values for the Olivetti Heart Study were transformed as published previously. NT-proBNP measurements that were provided in pmol/L were converted to pg/mL by multiplying values by a factor of 8·457.

eTable 2. Assessment of prevalent and incident cardiovascular disease in the 40 cohorts contributing to the NPSC.

Study design / study acronym	Ascertainment of prevalent diseases					Ascertainment of incident outcomes				Classification of incident outcomes							
										MI			Stroke				
	CHD	Revasc	Heart failure	Stroke	Diabetes	Death	Non-fatal MI	Non-fatal stroke	Non-fatal heart failure	Definite	Probable	Silent	Ischemic	Hemo-rrhagic	SAH	Unclassified	TIAs included?
**Prospective cohort study**																	
ARIC	+	++	++	+	++	**	++	++	++	✓	✓	o	✓	✓	✓	✓	o
ATTICA	++	NS	NS	++	++	*	+	NS	NA	NS	NS	NS	o	o	o	✓	o
BRHS	+	-	+	+	++	**	++	NS	NA	✓	o	o	o	o	o	✓	NS
BRUN	++	++	++	++	++	**	++	++	NA	✓	o	o	✓	✓	o	o	✓
CHS	++	++	++	++	++	**	++	++	++	✓	o	o	✓	✓	o	✓	NS
COPEN	++	++	++	++	++	*	++	++	NA	✓	o	o	✓	✓	o	o	✓
DHS	++	++	++	++	++	**	++	++	++	✓	o	o	✓	✓	✓	✓	o
FINRISK97	++	++	-	++	+	**	++	++	++	✓	o	o	✓	✓	✓	✓	o
FRAMOFF	++	++	++	++	++	**	++	++	++	✓	o	✓	✓	✓	✓	o	o
HISAYAMA	++	++	++	++	++	**	++	++	NA	✓	o	✓	✓	✓	o	o	o
KIHD	++	++	++	++	++	**	++	++	NA	✓	o	o	✓	✓	o	o	o
KISTORP	+	-	+	+	+	*	++	+	+	✓	o	o	o	o	o	✓	✓
LIFE	++	++	++	++	++	**	++	++	++	NS	NS	NS	✓	✓	o	✓	o
MDCS	++	-	++	++	++	**	NS	++	++	NS	NS	NS	✓	✓	✓	✓	o
MESA	+	+	+	+	++	**	++	++	NA	✓	NS	✓	✓	✓	✓	✓	NS
MONICA/KORA3	+	-	-	+	+	**	++	+	NA	✓	o	o	✓	✓	✓	✓	✓
OHS	+	+	-	+	++	NA	NA	NA	NA	NA	NA	NA	NA	NA	NA	NA	NA
PIVUS	+	+	++	+	++	NA	++	++	NA	✓	o	o	o	o	o	✓	o
PREVEND	++	-	-	++	++	**	NS	NS	++	NS	NS	NS	NS	NS	NS	NS	NS
PRIME	++	+	-	++	++	**	++	++	NA	✓	o	o	✓	✓	✓	✓	o
PTLBNP	++	++	++	++	++	*	NA	NA	NA	NA	NA	NA	NA	NA	NA	NA	NA
PROSPER	++	++	++	++	+	**	++	++	++	✓	o	o	o	o	o	✓	o
RANCHO	++	++	+	++	++	*	++	++	+	✓	o	o	✓	✓	o	✓	o
REYKOFF	++	++	-	++	++	*	++	NA	NA	✓	✓	o	NA	NA	NA	NA	NA
RS-I	++	++	++	++	++	**	++	++	NA	✓	o	o	✓	✓	✓	✓	NS
RS-II	++	++	++	++	++	**	++	++	NA	✓	o	o	✓	✓	✓	✓	NS
SHS	++	++	++	++	++	**	++	++	++	✓	o	o	✓	✓	✓	✓	o
ULSAM	++	++	++	++	++	**	++	++	++	✓	o	o	✓	✓	✓	✓	o
WOSCOPS	++	++	++	++	++	**	++	++	NA	✓	✓	o	o	o	o	✓	NS
**Case-cohort study**																	
DANMON	+	+	+	+	+ / -	*	++	++	++ / NA	✓	o	o	✓	✓	✓	✓	o
REGARDS	+	-	-	+	+	**	++	++	NA	✓	o	o	✓	✓	o	✓	o
WHIOS	+	+	+	+	+	**	++	++	NA	✓	o	o	✓	✓	o	o	o
WHS	+	+	-	+	+	**	++	++	NA	✓	o	o	✓	✓	✓	✓	o
**Nested case-control study**																	
BWHHS	+	++	-	+	+	**	++	NA	NA	✓	o	o	NA	NA	NA	NA	NA
NSHDS	+	-	-	+	++	*	++	NA	NA	✓	o	o	NA	NA	NA	NA	NA

Full study names are listed in the **eAppendix 5**. CHS consists of the cohorts CHS1 and CHS2. DANMON consists of the cohorts 45yr1936, 70yr1914, GenMon, Monica2, and Monica3. Abbreviations: -, not recorded; +, self-report only; ++, self-report supplemented by objective criteria (ECG, echocardiography, enzymes, imaging); *, based on death certificate only; **, based on death certificate supplemented by medical record; ✓, feature included in criteria; o, feature not included in criteria; NA, not available; NS, not stated; Revasc, coronary revascularization; SAH, subarachnoid haemorrhage; TIA, transient ischemic attack.

eTable 3. Baseline characteristics of participants in the 40 cohorts contributing to the NPSC.

Study design / study acronym	Total participants	Age (yrs) mean (SD)	Female (%)	History of diabetes (%)	Current smoking (%)	Systolic blood pressure (mmHg) mean (SD)	Total cholesterol (mmol/l) mean (SD)	HDL-cholesterol (mmol/l) mean (SD)	C-reactive protein (mg/l) median (25th, 75th percentile)	NT-proBNP (pg/ml) median (25th, 75th percentile)	BNP (pg/mL) median (25th, 75th percentile)
**Prospective cohort study**											
ARIC	8092	63 (6)	4650 (57)	1418 (18)	1145 (14)	127 (19)	5·2 (0·9)	1·32 (0·43)	2·24 [1·03, 5·08]	61 [30, 115]	-
ATTICA	392	41 (12)	195 (50)	11 (3)	166 (43)	121 (17)	5·0 (1·1)	1·26 (0·36)	0·97 [0·49, 2·09]	28 [16, 47]	-
BRHS	3122	68 (5)	0 (0)	197 (6)	375 (16)	149 (24)	6·1 (1·1)	1·33 (0·34)	1·48 [0·79, 3·25]	81 [42, 164]	-
BRUN	783	58 (11)	405 (52)	32 (4)	192 (25)	145 (22)	5·7 (1·0)	1·48 (0·38)	1·44 [0·84, 2·76]	80 [44, 136]	-
CHS	3888	73 (5)	2459 (63)	583 (15)	445 (11)	136 (21)	5·5 (1·0)	1·44 (0·40)	2·38 [1·19, 4·38]	100 [53, 187]	-
COPEN	4864	58 (16)	2920 (60)	187 (4)	1603 (33)	138 (23)	5·5 (1·1)	1·50 (0·48)	1·52 [0·72, 3·57]	152 [76, 262]	-
DHS	2692	44 (10)	1524 (57)	263 (10)	473 (19)	124 (18)	4·7 (1·0)	1·29 (0·38)	2·70 [1·10, 6·50]	27 [13, 56]	9 [3, 19]
FINRISK97	7717	47 (13)	3948 (51)	211 (3)	2061 (27)	136 (20)	5·5 (1·1)	1·40 (0·36)	1·50 [0·70, 3·40]	42 [20, 79]	14 [7, 26]
FRAMOFF	3008	58 (10)	1670 (56)	232 (8)	63 (2)	128 (19)	5·4 (1·0)	1·34 (0·42)	1·98 [0·87, 4·54]	-	8 [4, 16]
HISAYAMA	3104	62 (12)	1801 (58)	311 (10)	685 (22)	131 (21)	5·3 (0·9)	1·62 (0·42)	-	52 [26, 99]	-
KIHD	788	55 (7)	0 (0)	37 (5)	216 (27)	135 (17)	5·5 (0·9)	1·12 (0·29)	1·35 [0·71, 2·85]	123 [90, 190]	-
KISTORP	574	67 (11)	335 (58)	30 (5)	213 (37)	144 (23)	5·8 (1·1)	-	2·28 [1·02, 4·82]	243 [140, 463]	21 [15, 32]
LIFE	731	67 (7)	420 (57)	66 (9)	117 (16)	174 (13)	6·1 (1·2)	1·50 (0·41)	-	150 [82, 287]	-
MDCS	5010	57 (6)	2999 (60)	402 (8)	1304 (27)	141 (19)	6·2 (1·1)	1·39 (0·37)	1·30 [0·70, 2·80]	61 [34, 110]	-
MESA	5687	62 (10)	2927 (51)	722 (13)	818 (14)	126 (21)	5·0 (0·9)	1·31 (0·38)	1·85 [0·82, 4·18]	55 [24, 113]	-
MONICA/KORA3	1310	50 (14)	652 (50)	52 (4)	326 (25)	134 (20)	6·0 (1·1)	1·40 (0·43)	1·28 [0·60, 2·59]	53 [31, 98]	8 [4, 14]
OHS	847	59 (7)	0 (0)	42 (100)	294 (35)	139 (15)	5·6 (1·0)	1·21 (0·30)	1·60 [0·94, 2·83]	183 [132, 258]	-
PIVUS	845	70 (0)	448 (53)	65 (8)	88 (10)	150 (23)	5·5 (1·0)	1·54 (0·42)	1·21 [0·62, 2·32]	103 [61, 160]	39 [23, 66]
PREVEND	6767	49 (12)	3437 (51)	240 (4)	2295 (34)	129 (20)	5·7 (1·1)	1·33 (0·40)	1·24 [0·55, 2·83]	36 [16, 69]	-
PRIME	2327	55 (3)	0 (0)	29 (1)	733 (31)	134 (20)	5·9 (1·0)	1·19 (0·33)	1·61 [0·80, 3·13]	30 [16, 53]	17 [11, 28]
PROSPER	2964	76 (3)	1731 (58)	362 (12)	921 (31)	156 (22)	5·0 (1·1)	1·40 (0·39)	2·22 [1·09, 4·30]	127 [70, 235]	-
PTLBNP	4680	70 (14)	2810 (60)	248 (100)	-	-	-	-	-	139 [59, 368]	-
RANCHO	711	76 (9)	474 (67)	52 (7)	28 (4)	137 (21)	5·4 (0·9)	1·60 (0·49)	-	149 [62, 297]	-
REYKOFF	3723	47 (9)	1930 (52)	153 (4)	1197 (33)	127 (16)	5·7 (1·1)	1·37 (0·38)	1·30 [0·70, 2·80]	33 [14, 64]	-
RS–I	2740	72 (7)	1677 (61)	364 (13)	435 (16)	143 (21)	5·9 (1·0)	1·43 (0·40)	2·27 [1·16, 4·35]	86 [49, 155]	-
RS–II	2127	64 (7)	1192 (56)	206 (10)	506 (24)	143 (21)	5·8 (1·0)	1·39 (0·37)	0·90 [0·30, 2·30]	57 [31, 110]	-
SHS	475	60 (8)	293 (62)	207 (44)	201 (43)	126 (21)	5·0 (1·0)	1·11 (0·36)	3·70 [1·90, 6·60]	-	16 [9, 32]
ULSAM	925	71 (1)	0 (0)	108 (12)	194 (21)	147 (18)	5·8 (1·0)	1·30 (0·34)	1·80 [0·91, 3·80]	92 [54, 163]	-
WOSCOPS	4585	55 (6)	0 (0)	50 (1)	1942 (42)	135 (17)	7·1 (0·6)	1·14 (0·24)	1·76 [0·83, 3·62]	30 [14, 56]	-
**Case–cohort study**											
DANMON	1274	56 (12)	533 (42)	56 (5)	656 (52)	134 (22)	6·4 (1·2)	1·39 (0·39)	1·59 [0·74, 3·80]	67 [30, 147]	-
REGARDS	1605	67 (11)	778 (48)	319 (21)	272 (17)	131 (18)	5·0 (1·0)	1·32 (0·43)	2·43 [1·03, 5·16]	84 [39, 191]	-
WHIOS	3363	67 (7)	3363 (100)	237 (7)	225 (7)	132 (18)	5·9 (1·2)	1·41 (0·44)	2·62 [1·15, 5·45]	104 [61, 181]	-
WHS	1541	59 (8)	1541 (100)	512 (33)	221 (14)	131 (15)	5·7 (1·1)	1·29 (0·38)	3·13 [1·37, 6·07]	64 [35, 114]	-
**Nested case–control study**											
BWHHS	1007	70 (5)	1007 (100)	57 (6)	125 (12)	150 (25)	6·7 (1·2)	1·65 (0·43)	1·72 [0·86, 3·84]	143 [82, 250]	-
NSHDS	1349	55 (8)	409 (30)	35 (3)	340 (26)	136 (17)	6·3 (1·3)	1·25 (0·34)	1·52 [0·78, 3·05]	43 [22, 87]	-
**TOTAL**	95617	61 (10)	48528 (51)	8096 (9)	20875 (23)	137 (20)	5·7 (1·0)	1·36 (0·39)	1·76 [0·81, 3·92]	64 [30, 135]	13 [6, 25]

Full study names are listed in the **eAppendix 5**. CHS consists of the cohorts CHS1 and CHS2. DANMON consists of the cohorts 45yr1936, 70yr1914, GenMon, Monica2, and Monica3. Abbreviations: SD, standard deviation.

eTable 4. Available follow-up information for participants included in the NPSC.

Study design / study acronym	Total participants	Duration of follow-up, median (interquartile range)	Person-years of follow-up	CHD	Stroke	Heart failure
**Prospective cohort study**						
ARIC	8092	13·7 (10·0 to 14·7)	96693	316	343	638
ATTICA	392	5·7 (0·0 to 5·7)	1602	10	3	0
BRHS	3122	5·7 (5·2 to 6·3)	17182	135	27*	0
BRUN	783	20·2 (13·5 to 20·5)	13068	61	56	9*
CHS	3888	9·6 (6·3 to 12·3)	34729	459	390	450
COPEN	4864	6·6 (6·0 to 7·1)	30441	105	193	0
DHS	2692	6·4 (6·0 to 6·9)	17031	27	26	24
FINRISK97	7717	11·8 (11·8 to 11·9)	86272	106	103	478
FRAMOFF	3008	11·8 (10·9 to 12·7)	33821	116	72	60
HISAYAMA	3104	5·2 (5·2 to 5·2)	15506	36	80	3*
KIHD	788	15·8 (12·4 to 16·7)	10941	113	47	1*
KISTORP	574	4·9 (4·6 to 5·1)	2573	9	24	17
LIFE	731	4·5 (4·4 to 4·5)	3104	20	20	10
MDCS	5010	15·7 (14·9 to 16·4)	72926	300	264	89
MESA	5687	8·5 (7·6 to 8·6)	43074	117	118	0
MONICA/KORA3	1310	14·0 (13·3 to 14·3)	16755	70	14*	2*
OHS	847	-	0	0	0	0
PIVUS	845	4·8 (4·7 to 5·0)	3969	22	30	0
PREVEND	6767	10·6 (10·2 to 10·9)	65278	171	39	141
PRIME	2327	18·0 (18·0 to 18·0)	37612	160	90	1*
PROSPER	2964	2·8 (2·5 to 3·0)	7903	175	91	61
PTLBNP	4680	3·1 (2·5 to 3·8)	14319	0	0	0
RANCHO	711	8·7 (6·0 to 9·4)	5374	39	28	19
REYKOFF	3723	7·1 (5·9 to 7·8)	25332	39	0	0
RS-I	2740	5·8 (5·2 to 6·7)	15309	74	120	10*
RS-II	2127	10·1 (9·5 to 10·5)	19934	75	53	8*
SHS	475	12·2 (5·7 to 13·4)	4545	76	27	39
ULSAM	925	13·4 (7·3 to 15·2)	10320	124	97	96
WOSCOPS	4585	5·0 (4·5 to 5·5)	22110	187	52	0
**Case-cohort study**						
DANMON	1274	14·9 (6·9 to 18·8)	16753	434	103	56
REGARDS	1605	4·2 (2·0 to 6·0)	6548	495	391	0
WHIOS	3363	8·7 (5·4 to 10·8)	26339	675	824	0
WHS	1541	12·2 (8·6 to 13·5)	16390	192	277	0
**Nested case-control study**						
BWHHS	1007	12·1 (8·9 to 12·7)	10459	82	0	0
NSHDS	1349	3·6 (2·0 to 5·6)	5312	480*	0	0
**TOTAL**	95617	7·8 (5·2 to 11·8)	809525	5500	4002	2212

Full study names are listed in the **eAppendix 5**. CHS consists of the cohorts CHS1 and CHS2. DANMON consists of the cohorts 45yr1936, 70yr1914, GenMon, Monica2, and Monica3. *Study provided information on fatal outcomes only.

eTable 5. Cross-sectional association of NT-proBNP with other baseline variables.

Variable	No. of cohorts / participants	Mean (SD) or %	Age- and sex-adjusted % mean difference (95% CI) in NT-proBNP per SD or compared to reference group
**Natriuretic peptides**			
Log NT-proBNP (pg/ml)	38 / 87626	4·3 (1·1)	–
Log BNP (pg/mL)	6 / 12745	2·7 (1)	102% (67, 144)***
**Questionnaire based**			
Age at survey (yrs)	38 / 87626	61 (9·7)	54% (46, 64)***
Sex			
Male	35 / 43958	50·2%	[Reference]
Female	32 / 43668	49·8%	55% (42, 70)***
Ethnicity			
White	21 / 45577	83·8%	[Reference]
Non-white	13 / 8838	16·2%	-27% (-34, -20)***
Current smoker			
No	37 / 61991	76·1%	[Reference]
Yes	37 / 19521	23·9%	7% (4, 10)***
Alcohol drinker			
No	29 / 31374	50·2%	[Reference]
Yes	24 / 31089	49·8%	5% (2, 8)**
**Baseline history of disease**			
History of hypertension			
No	20 / 35757	61·6%	[Reference]
Yes	25 / 22298	38·4%	23% (15, 31)***
History of diabetes			
No	35 / 74690	91·2%	[Reference]
Yes	37 / 7236	8·8%	-7% (-12, -2)**
**Medication use**			
Statins			
No	18 / 35324	96·5%	[Reference]
Yes	12 / 1272	3·5%	-10% (-18, -1)*
Anti-diabetic			
No	22 / 41572	94·6%	[Reference]
Yes	21 / 2376	5·4%	-1% (-7, 5)
Anti-hypertensive			
No	28 / 39012	70·6%	[Reference]
Yes	24 / 16253	29·4%	23% (16, 30)***
**Physical measurements**			
SBP (mmHg)	37 / 82641	138 (20)	11% (9, 14)***
DBP (mmHg)	36 / 79682	81 (10)	2% (0, 3)*
BMI (kg/m^2)	34 / 76294	27 (4·5)	-7% (-9, -6)***
Waist/hip ratio	25 / 62450	·9 (·082)	-8% (-10, -6)***
**Lipids**			
Total cholesterol (mmol/l)	37 / 82463	5·7 (1)	-10% (-12, -8)***
Non-HDL-C (mmol/l)	36 / 81173	4·3 (1)	-10% (-12, -9)***
HDL-C (mmol/l)	36 / 81219	1·4 (·39)	4% (2, 5)***
Log triglycerides (mmol/l)	31 / 71403	·28 (·51)	-8% (-10, -7)***
**Renal function**			
Creatinine (μmol/l)	24 / 56803	82 (23)	13% (9, 17)***
Estimated GFR	24 / 56803	80 (15)	-9% (-11, -6)***
**Dysglycaemia and inflammation**			
Log Fasting glucose (mmol/l)	15 / 31343	1·7 (·18)	-5% (-7, -3)***
Log CRP (mg/l)	32 / 70469	·54 (1·1)	6% (3, 8)***

To avoid over-sampling of incident cases, the analysis was restricted to the random subcohort for case-cohort studies and to controls for nested case-control studies. Abbreviations: BMI, body mass index; estimated GFR, estimated glomerular filtration rate (using the CKD-EPI 2009 formula); CRP, C-reactive protein; DBP, diastolic blood pressure; HDL-C, high-density lipoprotein cholesterol; Non-HDL-C, non high-density lipoprotein cholesterol; SBP, systolic blood pressure; SD, standard deviation. *P<0.05, **P<0.01, and ***P<0.001.

eTable 6. Cross-sectional association of BNP with other baseline variables.

Variable	No. of cohorts / participants	Mean (SD) or %	Age- and sex-adjusted % mean difference (95% CI) in BNP per SD or compared to reference group
**Natriuretic peptides**			
Log BNP (pg/mL)	8 / 16753	2·7 (1)	–
**Questionnaire based**			
Age at survey (yrs)	9 / 16753	56 (11)	35% (27, 43)***
Sex			
Male	8 / 8614	51·4%	[Reference]
Female	7 / 8139	48·6%	34% (18, 51)***
Ethnicity			
White	4 / 11719	88·0%	[Reference]
Non-white	2 / 1597	12·0%	-0% (-26, 34)
Current smoker			
No	8 / 13030	78·7%	[Reference]
Yes	8 / 3535	21·3%	3% (-3, 9)
Alcohol drinker			
No	6 / 6092	39·8%	[Reference]
Yes	5 / 9222	60·2%	5% (-6, 18)
**Baseline history of disease**			
History of hypertension			
No	6 / 8523	67·0%	[Reference]
Yes	6 / 4205	33·0%	11% (3, 19)**
History of diabetes			
No	8 / 15695	94·2%	[Reference]
Yes	8 / 971	5·8%	2% (-8, 13)
**Medication use**			
Statins			
No	4 / 9867	99·3%	[Reference]
Yes	3 / 74	0·7%	-16% (-34, 8)
Anti-diabetic			
No	6 / 7076	93·4%	[Reference]
Yes	6 / 496	6·6%	5% (-7, 18)
Anti-hypertensive			
No	8 / 8108	74·5%	[Reference]
Yes	8 / 2782	25·5%	21% (10, 34)***
**Physical measurements**			
SBP (mmHg)	8 / 16727	134 (20)	8% (5, 10)***
DBP (mmHg)	8 / 16718	80 (11)	-1% (-5, 3)
BMI (kg/m^2)	6 / 14499	27 (4·5)	-5% (-6, -3)***
Waist/hip ratio	6 / 14439	·91 (·086)	-7% (-11, -4)***
**Lipids**			
Total cholesterol (mmol/l)	8 / 16723	5·5 (1)	-8% (-11, -5)***
Non-HDL-C (mmol/l)	7 / 16186	4·1 (1·1)	-10% (-13, -7)***
HDL-C (mmol/l)	7 / 16188	1·3 (·38)	4% (3, 6)***
Log triglycerides (mmol/l)	7 / 16199	·3 (·54)	-9% (-12, -6)***
**Renal function**			
Creatinine (µmol/l)	7 / 9404	84 (31)	8% (4, 13)***
Estimated GFR	7 / 9404	82 (16)	-3% (-5, -1)**
**Dysglycaemia and inflammation**			
Log Fasting glucose (mmol/l)	4 / 5317	1·7 (·22)	-2% (-5, 1)
Log CRP (mg/l)	8 / 10236	·68 (1·1)	-0% (-3, 3)

To avoid over-sampling of incident cases, the analysis was restricted to the random subcohort from case-cohort studies and to controls for nested case-control studies. Abbreviations: BMI, body mass index; estimated GFR, estimated glomerular filtration rate (using the CKD-EPI 2009 formula); CRP, C-reactive protein; DBP, diastolic blood pressure; HDL-C, high-density lipoprotein cholesterol; Non-HDL-C, non high-density lipoprotein cholesterol; SBP, systolic blood pressure; SD, standard deviation. *P<0.05, **P<0.01, and ***P<0.001.

eTable 7. Risk ratios for individual cardiovascular outcomes comparing people in the top third versus the bottom third of NT-proBNP levels.

Level of adjustment	CHD		Stroke		Heart failure	
	No. of cohorts / participants / events	Risk ratio (95% CI) top vs. bottom third	No. of cohorts / participants / events	Risk ratio (95% CI) top vs. bottom third	No. of cohorts / participants / events	Risk ratio (95% CI) top vs. bottom third
**Basic adjustment**						
Age and sex	34 / 82015 / 4716	1·69 (1·44, 1·97)	30 / 76839 / 3768	1·97 (1·70, 2·29)	16 / 42128 / 2021	3·73 (2·80, 4·97)
Plus non-blood based markers*		1·51 (1·31, 1·75)		1·77 (1·54, 2·02)		3·43 (2·65, 4·43)
Plus lipid markers†		1·67 (1·45, 1·93)		1·81 (1·58, 2·07)		3·45 (2·66, 4·46)
**Additional adjustment**						
Basic adjustment‡	32 / 76425 / 4502	1·69 (1·46, 1·96)	28 / 71289 / 3647	1·78 (1·55, 2·05)	14 / 36629 / 1938	3·11 (2·47, 3·91)
Plus body mass index		1·70 (1·46, 1·98)		1·76 (1·54, 2·03)		3·26 (2·60, 4·09)
Basic adjustment‡	29 / 69321 / 4283	1·65 (1·43, 1·91)	26 / 64548 / 3487	1·72 (1·50, 1·97)	13 / 33429 / 1606	3·88 (2·82, 5·35)
Plus log C-reactive protein		1·60 (1·38, 1·84)		1·69 (1·48, 1·93)		3·68 (2·70, 5·00)
Basic adjustment‡	28 / 69845 / 3666	1·67 (1·42, 1·97)	25 / 65117 / 2674	1·72 (1·47, 2·01)	13 / 35883 / 1944	3·43 (2·61, 4·51)
Plus log triglycerides		1·68 (1·42, 1·98)		1·72 (1·47, 2·02)		3·42 (2·61, 4·49)
Basic adjustment‡	26 / 55777 / 3514	1·60 (1·36, 1·89)	23 / 51083 / 3155	1·80 (1·52, 2·15)	11 / 24880 / 1350	3·39 (2·55, 4·51)
Plus anti-hypertensive medication		1·58 (1·33, 1·87)		1·79 (1·51, 2·13)		3·25 (2·49, 4·26)
Basic adjustment‡	25 / 62629 / 3003	1·67 (1·38, 2·02)	21 / 57539 / 2275	1·66 (1·40, 1·98)	9 / 27567 / 1393	3·83 (2·59, 5·65)
Plus fasting glucose		1·67 (1·38, 2·02)		1·70 (1·42, 2·03)		3·97 (2·74, 5·74)
Basic adjustment‡	21 / 55224 / 2733	1·70 (1·40, 2·07)	19 / 50627 / 2202	1·76 (1·54, 2·02)	9 / 25979 / 1365	3·69 (2·44, 5·59)
Plus eGFR		1·69 (1·39, 2·05)		1·74 (1·52, 2·00)		3·71 (2·43, 5·68)
Basic adjustment‡	21 / 55464 / 3245	1·61 (1·31, 1·98)	18 / 50507 / 2928	1·89 (1·66, 2·16)	11 / 36077 / 1879	3·46 (2·59, 4·63)
Plus ethnicity		1·61 (1·32, 1·96)		1·94 (1·69, 2·23)		3·65 (2·73, 4·88)
Basic adjustment‡	14 / 37445 / 1932	1·56 (1·33, 1·83)	13 / 36459 / 1920	2·08 (1·73, 2·51)	5 / 13876 / 816	3·54 (2·32, 5·39)
Plus lipid-lowering medication		1·56 (1·33, 1·83)		2·08 (1·72, 2·51)		3·52 (2·30, 5·39)

Abbreviation: eGFR, estimated glomerular filtration rate (using the CKD-EPI 2009 formula). *History of diabetes, smoking status, and systolic blood pressure. †Total cholesterol and HDL-cholesterol. ‡Adjusted for age, sex, non-blood based and lipid markers.

eTable 8. Sensitivity analyses using study-specific distribution to define thirds or 1-SD higher log NT-proBNP to calculate risk ratios for individual cardiovascular outcomes.

	NT-proBNP	HDL-cholesterol
**RR (95% CI)**		
Thirds defined overall	*Top vs. bottom*	*Bottom vs. top*
CHD	1·67 (1·45, 1·93)	1·87 (1·67, 2·08)
Stroke	1·81 (1·58, 2·07)	1·35 (1·14, 1·60)
Heart failure	3·45 (2·66, 4·46)	1·28 (1·08, 1·53)
Thirds defined within studies	*Top vs. bottom*	*Bottom vs. top*
CHD	1·61 (1·41, 1·83)	1·85 (1·68, 2·05)
Stroke	1·73 (1·51, 1·98)	1·39 (1·16, 1·66)
Heart failure	3·59 (2·83, 4·56)	1·38 (1·21, 1·58)
Per 1 SD	*1 SD higher*	*1 SD lower*
CHD	1·33 (1·25, 1·42)	1·33 (1·25, 1·41)
Stroke	1·43 (1·34, 1·53)	1·16 (1·07, 1·26)
Heart failure	2·07 (1·82, 2·36)	1·17 (1·09, 1·26)

*defined as a non-fatal myocardial infarction or fatal CHD or any fatal or non-fatal stroke. Adjusted for age, smoking status, history of diabetes, systolic blood pressure, total cholesterol and HDL-cholesterol, and stratified by sex.

eTable 9. Risk ratios for individual cardiovascular outcomes comparing people in the top third versus the bottom third of BNP levels.

Level of adjustment	CHD		Stroke		Heart failure	
	No. of cohorts / participants / events	Risk ratio (95% CI) top vs. bottom third	No. of cohorts / participants / events	Risk ratio (95% CI) top vs. bottom third	No. of cohorts / participants / events	Risk ratio (95% CI) top vs. bottom third
**Basic adjustment**						
Age and sex	7 / 15909 / 507	1·09 (0·83, 1·43)	7 / 15909 / 312	1·14 (0·83, 1·57)	4 / 12202 / 563	2·31 (1·45, 3·67)
Plus non-blood based markers*		1·06 (0·79, 1·42)		1·07 (0·78, 1·48)		2·21 (1·43, 3·42)
Plus lipid markers†		1·16 (0·88, 1·54)		1·10 (0·79, 1·53)		2·23 (1·44, 3·46)
**Additional adjustment**						
Basic adjustment‡	6 / 14353 / 488	1·21 (0·88, 1·65)	6 / 14353 / 292	1·08 (0·77, 1·53)	3 / 10652 / 545	1·94 (1·28, 2·95)
Plus body mass index		1·22 (0·89, 1·67)		1·08 (0·77, 1·53)		2·00 (1·27, 3·16)
Basic adjustment‡	7 / 9496 / 434	1·12 (0·81, 1·55)	7 / 9496 / 254	1·04 (0·72, 1·51)	4 / 6030 / 240	2·63 (1·57, 4·39)
Plus log C-reactive protein		1·12 (0·81, 1·53)		1·04 (0·72, 1·51)		2·66 (1·58, 4·49)
Basic adjustment‡	7 / 15908 / 507	1·16 (0·88, 1·54)	7 / 15908 / 312	1·10 (0·79, 1·53)		2·23 (1·44, 3·46)
Plus log triglycerides		1·14 (0·87, 1·49)		1·11 (0·80, 1·55)	4 / 12202 / 563	2·29 (1·47, 3·59)
Basic adjustment‡	7 / 10202 / 446	1·16 (0·87, 1·56)	7 / 10202 / 261	1·01 (0·71, 1·45)	4 / 6495 / 356	2·03 (1·20, 3·43)
Plus anti-hypertensive medication		1·16 (0·87, 1·55)		0·99 (0·69, 1·43)		2·00 (1·17, 3·40)
Basic adjustment‡	6 / 8358 / 377	1·16 (0·80, 1·67)	6 / 8358 / 202	1·07 (0·72, 1·58)	3 / 4990 / 114	2·56 (1·20, 5·44)
Plus fasting glucose		1·18 (0·82, 1·69)		1·09 (0·73, 1·62)		2·73 (1·42, 5·26)
Basic adjustment‡	6 / 8663 / 399	1·17 (0·84, 1·64)	6 / 8663 / 214	1·03 (0·70, 1·52)	3 / 4974 / 114	2·56 (1·22, 5·40)
Plus eGFR		1·17 (0·82, 1·69)		1·01 (0·68, 1·48)		2·53 (1·22, 5·23)
Basic adjustment‡	5 / 13028 / 334	0·88 (0·63, 1·23)	5 / 13028 / 244	1·18 (0·81, 1·73)	4 / 12202 / 563	2·23 (1·44, 3·46)
Plus ethnicity		0·88 (0·63, 1·23)		1·20 (0·82, 1·74)		2·27 (1·44, 3·58)
Basic adjustment‡	4 / 9839 / 350	1·16 (0·76, 1·78)	4 / 9839 / 186	1·09 (0·73, 1·63)	3 / 8541 / 447	1·76 (1·06, 2·92)
Plus lipid-lowering medication		1·17 (0·76, 1·80)		1·08 (0·72, 1·63)		1·76 (1·06, 2·92)

Abbreviation: eGFR, estimated glomerular filtration rate (using the CKD-EPI 2009 formula). *History of diabetes, smoking status, and systolic blood pressure. †Total cholesterol and HDL-cholesterol. ‡Adjusted for age, sex, non-blood based and lipid markers.

eTable 10. Improvement in risk classification and integrated discrimination for first-onset composite cardiovascular disease outcomes by addition of information on NT-proBNP compared to that of HDL-cholesterol, for non-cases, cases, and overall.

Outcome / Model	Categorical NRI (95% CI) vs. preceding model using different cut-offs for clinical risk categories				Continuous NRI (95% CI)	IDI (95% CI) vs. preceding model
	ACC/AHA 2013	NICE 2014	ACCF/AHA 2010	ESC 2016*		
**CHD plus stroke**						
Conventional risk factors without HDL-C*	[Reference]	[Reference]	[Reference]	[Reference]	[Reference]	[Reference]
+ HDL-C						
Non-cases	0·001 (-0·003, 0·004)	0·001 (-0·003, 0·004)	-0·005 (-0·008, -0·003)***	-0·002 (-0·004, 0·000)	0·123 (0·103, 0·143)***	0·0005 (0·0002, 0·0007)***
Cases	0·008 (-0·000, 0·016)	0·013 (0·005, 0·022)**	0·023 (0·014, 0·032)***	0·015 (0·003, 0·027)*	0·170 (0·138, 0·201)***	0·0106 (0·0090, 0·0122)***
Overall	*0·009 (-0·000, 0·017)*	*0·014 (0·005, 0·023)***	*0·017 (0·008, 0·027)****	*0·014 (0·001, 0·026)**	*0·292 (0·252, 0·333)****	*0·0110 (0·0094, 0·0127)****
+ HDL-C + NT-proBNP						
Non-cases	0·029 (0·025, 0·032)***	0·025 (0·021, 0·028)***	0·012 (0·010, 0·014)***	0·030 (0·027, 0·033)***	0·174 (0·153, 0·195)***	0·0013 (0·0011, 0·0016)***
Cases	-0·001 (-0·009, 0·007)	0·002 (-0·006, 0·011)	0·014 (0·004, 0·024)**	-0·003 (-0·022, 0·015)	-0·020 (-0·053, 0·014)	0·0118 (0·0096, 0·0140)***
Overall	*0·027 (0·019, 0·036)****	*0·027 (0·018, 0·036)****	*0·026 (0·016, 0·036)****	*0·027 (0·008, 0·046)***	*0·154 (0·111, 0·198)****	*0·0131 (0·0109, 0·0154)****
**CHD plus stroke plus heart failure**						
Conventional risk factors without HDL-C*	[Reference]	[Reference]	[Reference]	[Reference]	[Reference]	[Reference]
+ HDL-C						
Non-cases	0·011 (0·008, 0·015)***	0·008 (0·005, 0·011)***	-0·003 (-0·005, -0·000)*	0·003 (0·001, 0·005)**	0·119 (0·105, 0·132)***	0·0003 (0·0001, 0·0005)**
Cases	0·006 (-0·001, 0·013)	0·009 (0·001, 0·017)*	0·018 (0·008, 0·029)***	-0·003 (-0·017, 0·012)	0·104 (0·071, 0·137)***	0·0041 (0·0031, 0·0052)***
Overall	*0·017 (0·009, 0·025)****	*0·017 (0·009, 0·026)****	*0·016 (0·005, 0·026)***	*0·000 (-0·014, 0·015)*	*0·223 (0·188, 0·258)****	*0·0044 (0·0033, 0·0055)****
+ HDL-C + NT-proBNP						
Non-cases	0·036 (0·032, 0·040)***	0·035 (0·031, 0·038)***	0·028 (0·025, 0·032)***	0·032 (0·029, 0·036)***	0·189 (0·176, 0·202)***	0·0026 (0·0023, 0·0030)***
Cases	-0·008 (-0·017, 0·001)	-0·009 (-0·019, 0·001)	0·003 (-0·010, 0·017)	0·005 (-0·018, 0·028)	0·008 (-0·025, 0·042)	0·0269 (0·0235, 0·0303)***
Overall	*0·028 (0·019, 0·038)****	*0·025 (0·015, 0·036)****	*0·032 (0·018, 0·046)****	*0·038 (0·015, 0·061)***	*0·198 (0·162, 0·234)****	*0·0295 (0·0261, 0·0329)****

*The reference model included information on age, sex, smoking, systolic blood pressure, history of diabetes, and levels of total cholesterol. The predicted 10-year CVD risk categories used to calculate the categorical NRIs were: <5%, 5% to <7·5%, and ≥7·5% according to the 2013 ACC/AHA guideline; <5%, 5% to <10%, and ≥10% according to the 2014 NICE guideline; <10%, 10% to <20%, and ≥20% according to the 2010 ACCF/AHA guideline,ⁱ and <2·5%, 2·5% to <5%, and ≥5% according to the 2012 ESC guideline. The analysis for ESC 2016 cutoffs involved data on 1557 fatal outcomes of CHD or stroke (from 21 cohorts with 39257 participants) and 1129 fatal outcomes of CHD, stroke or heart failure (from 17 cohorts with 35597 participants). The analyses for other cutoffs involved data on 4672 incident outcomes of CHD or stroke (from 19 cohorts with 37714 participants) and 4071 incident outcomes of CHD, stroke or heart failure (from 16 cohorts with 34822 participants). Abbreviations: ACC, American College of Cardiology; ACCF, American College of Cardiology Foundation; AHA, American Heart Association; ESC, European Society of Cardiology; IDI, integrated discrimination improvement; NICE, National Institute for Health and Care Excellence; NRI, net reclassification improvement.

eFigure 1. Box plots of study-specific distributions of natriuretic peptides grouped by assay type.

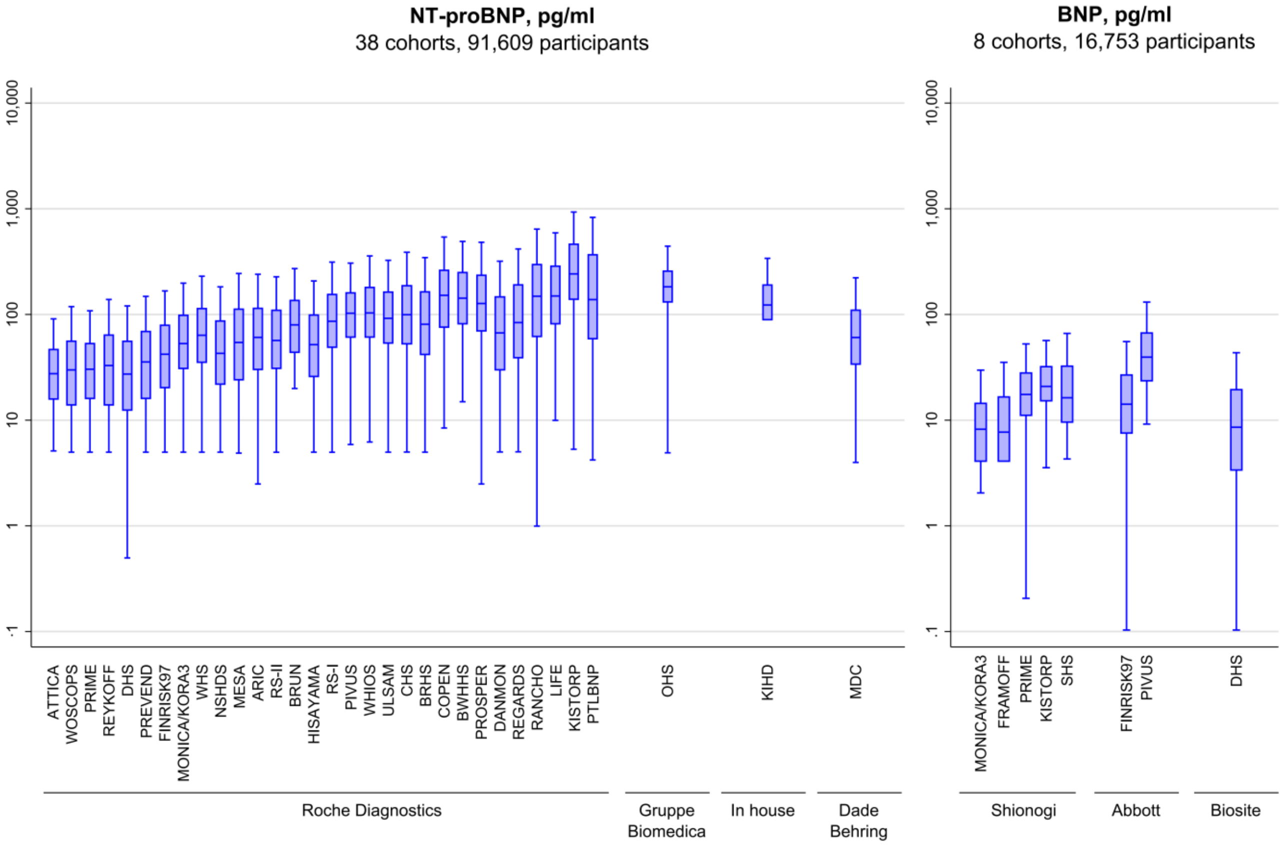

Full study names are listed in the **eAppendix 5**. CHS consists of the cohorts CHS1 and CHS2. DANMON consists of the cohorts 45yr1936, 70yr1914, GenMon, Monica2, and Monica3. The bottom and top of the box are the first and third quartiles. The end of the lower whisker is the lowest value within the 1·5 interquartile range of the lower quartile; the end of the higher whisker is the highest value within the 1·5 interquartile range of the upper quartile. Outliers are not shown.

eFigure 2. Association between NT-proBNP and BNP levels.

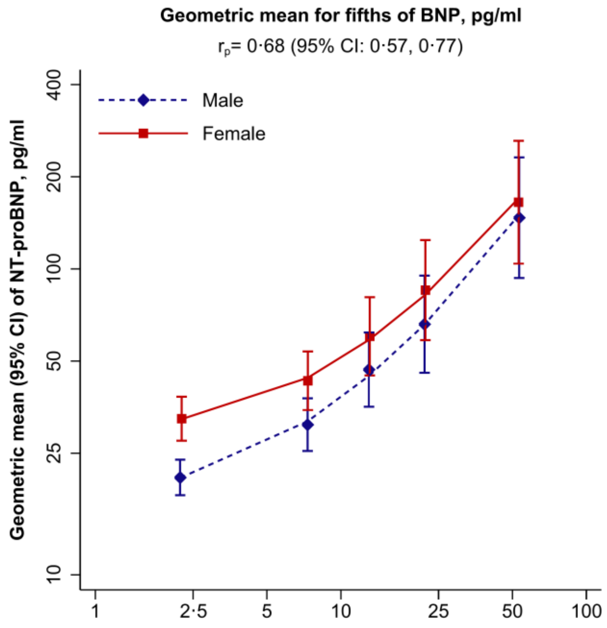

Response means were adjusted to age 60 years. To avoid over-sampling of incident cases, the analysis was restricted to the random subcohort for case-cohort studies and to controls for nested case-control studies. Abbreviation: r_p, partial correlation coefficient.

eFigure 3. Cross-sectional associations for males and females between NT-proBNP concentration and conventional risk factors.

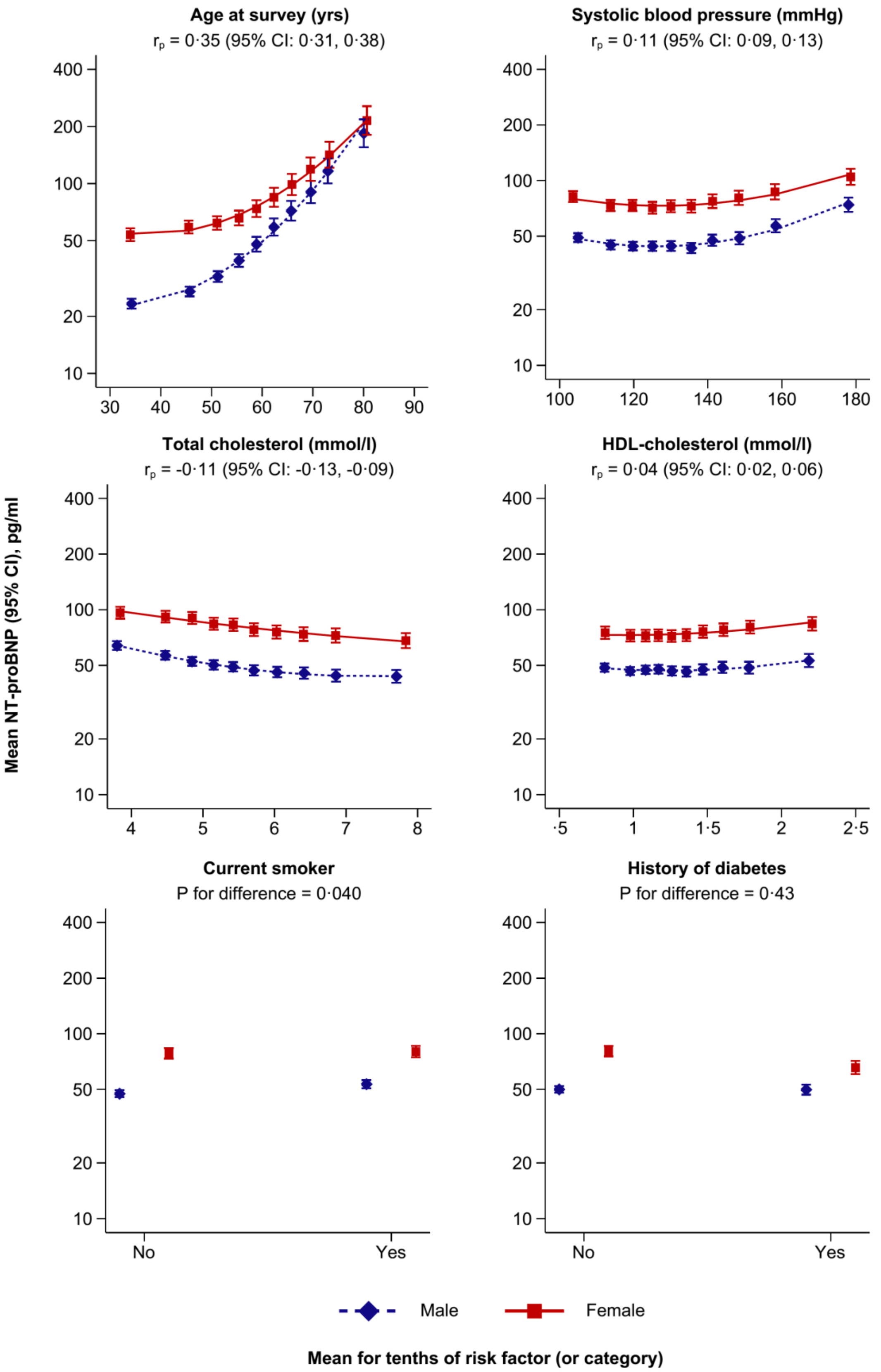

The geometric mean of NT-proBNP concentration was adjusted to age 60 years (except for age plot). The partial correlation coefficients r_p and P values for difference were adjusted for age and sex (except for the correlation coefficient with age, which was adjusted for sex only). To avoid over-sampling of incident cases, the analysis was restricted to the random subcohort for case-cohort studies and to controls for nested case-control studies.

eFigure 4. Study-specific risk ratios for individual cardiovascular outcomes comparing the top vs bottom third of NT-proBNP levels.

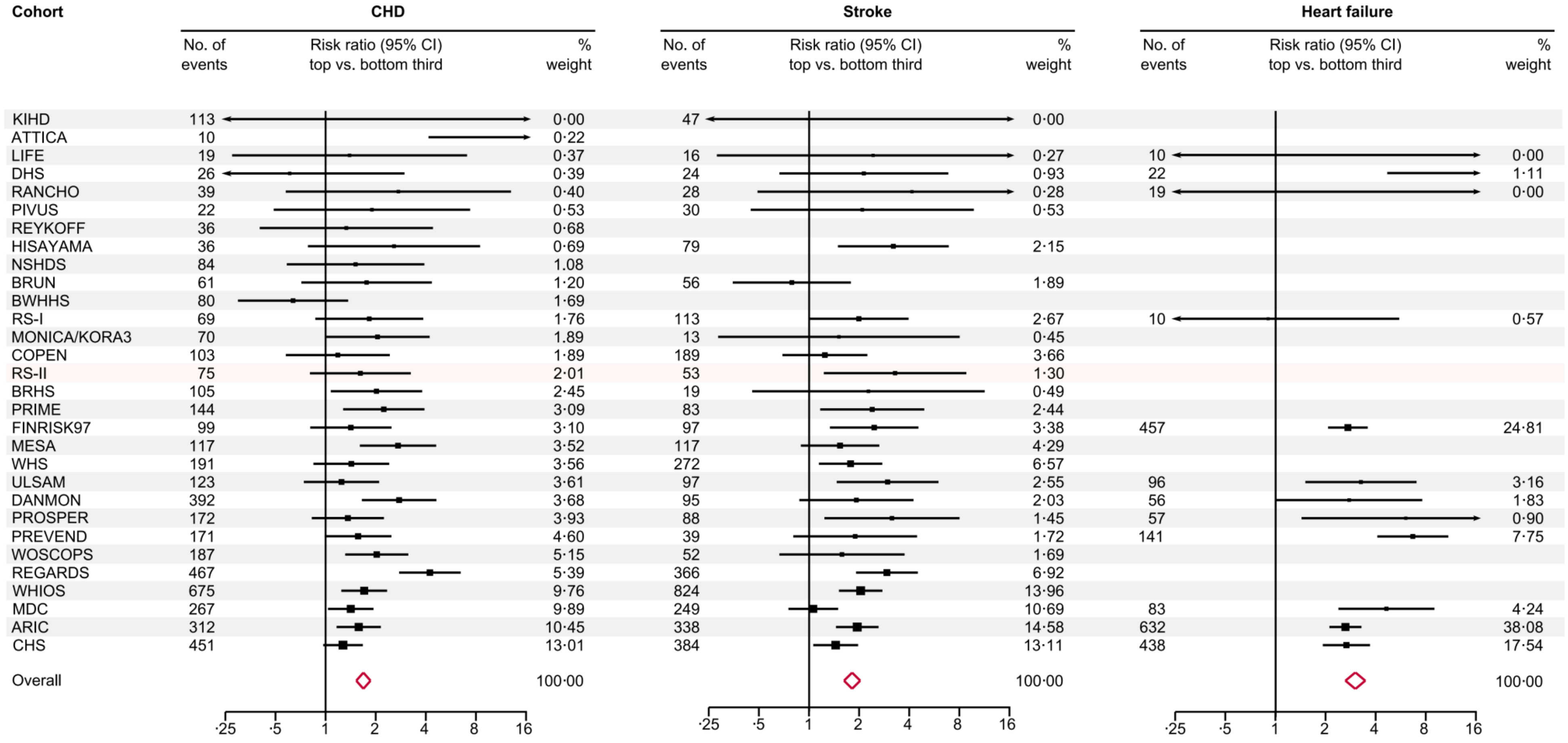

Full study names are listed in the **eAppendix 5**. CHS consists of the cohorts CHS1 and CHS2. DANMON consists of the cohorts 45yr1936, 70yr1914, GenMon, Monica2, and Monica3. Adjusted for age, smoking status, history of diabetes, systolic blood pressure, total cholesterol and HDL cholesterol, and stratified by sex. The I^2 values were 45% for CHD, 23% for stroke, and 54% for heart failure.

eFigure 5. Risk ratios for individual cardiovascular outcomes comparing the top vs bottom third of NT-proBNP levels across clinically relevant subgroups.

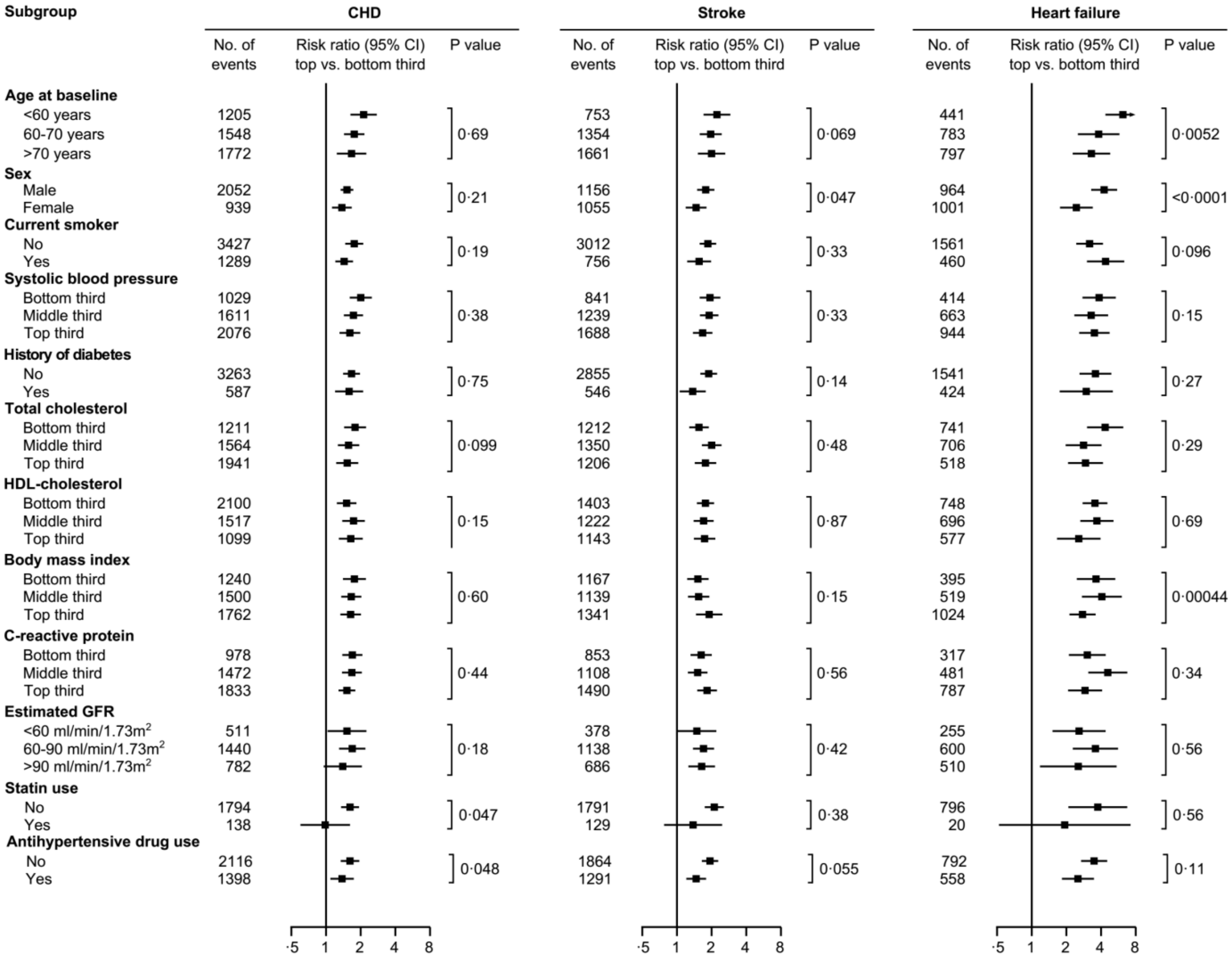

Adjusted for age, smoking status, history of diabetes, systolic blood pressure, total cholesterol and HDL-cholesterol, and stratified by sex. Abbreviations: estimated GFR, estimated glomerular filtration rate (using the CKD-EPI 2009 formula).

eFigure 6. Risk ratios for individual cardiovascular outcomes comparing the top vs bottom third of NT-proBNP levels according to different study-level characteristics.

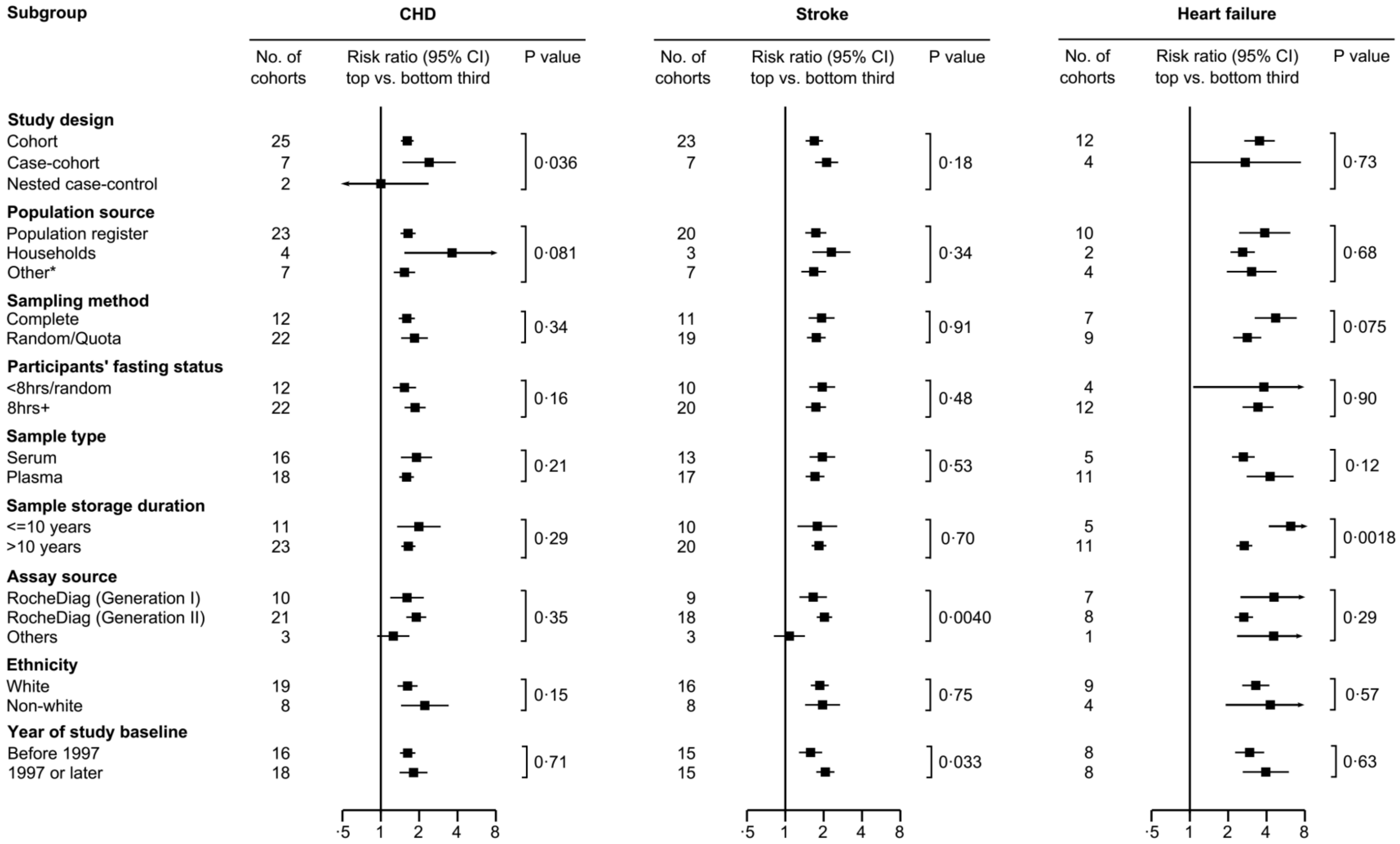

Adjusted for age, smoking status, history of diabetes, systolic blood pressure, total cholesterol and HDL cholesterol, and stratified by sex. Information on ethnicity was available in a subset of 27 cohorts with CHD outcomes, 24 cohorts with stroke outcomes, and 15 cohorts with heart failure outcomes. *Population recruited using occupational, general practitioner, or health service lists.

eFigure 7. Sensitivity analyses of the association of NT-proBNP levels with individual cardiovascular outcomes.

Endpoint / Analysis type	No. of cohorts	No. of events	Risk ratio (95% CI) top vs. bottom third
**CHD**			
Primary analysis	34	4716	1·67 (1·45, 1·93)
Defining thirds separately for males and females	34	4716	1·64 (1·43, 1·89)
Excluding people with high baseline NT-proBNP	34	4584	1·62 (1·41, 1·86)
Excluding first 5 years of follow-up	28	2307	1·54 (1·33, 1·79)
Studies with fatal and non-fatal outcomes	26	4236	1·71 (1·47, 1·98)
**Stroke**			
Primary analysis	30	3768	1·81 (1·58, 2·07)
Defining thirds separately for males and females	30	3768	1·79 (1·56, 2·05)
Excluding people with high baseline NT-proBNP	30	3670	1·73 (1·52, 1·96)
Excluding first 5 years of follow-up	22	1891	1·71 (1·45, 2·01)
Studies with fatal and non-fatal outcomes	23	3193	1·88 (1·59, 2·22)
**Heart failure**			
Primary analysis	16	2021	3·45 (2·66, 4·46)
Defining thirds separately for males and females	16	2021	3·09 (2·39, 3·99)
Excluding people with high baseline NT-proBNP	15	1911	2·84 (2·33, 3·47)
Excluding first 5 years of follow-up	11	1358	2·66 (2·10, 3·36)
Studies with fatal and non-fatal outcomes	9	852	3·59 (2·47, 5·24)

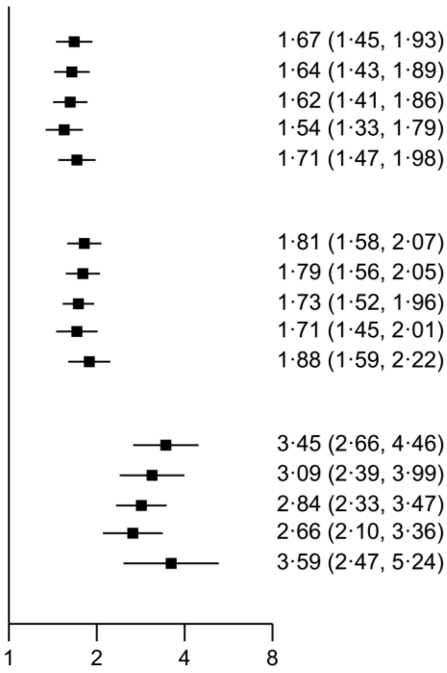

Adjusted for age, smoking status, history of diabetes, systolic blood pressure, total cholesterol and HDL cholesterol, and stratified by sex. The cutoff used to exclude people with high baseline levels of NT-proBNP were: >450 pg/ml for people aged 50 years or younger, >900 pg/ml for people aged 50-75 years, and >1800 pg/ml for people aged 75 years or older.

eFigure 8. Study-specific risk ratios for cardiovascular outcomes according to lengths of follow-up.

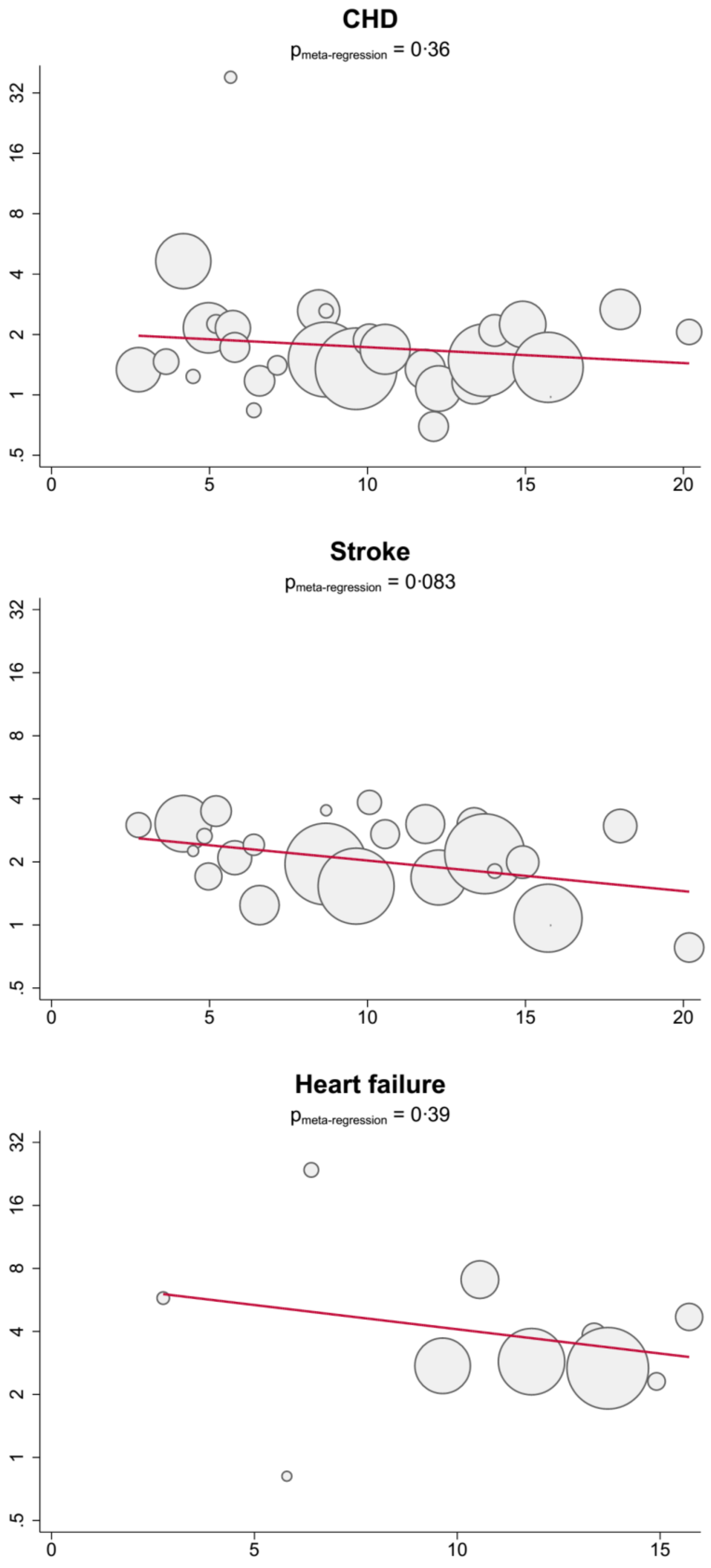

Adjusted for age, smoking status, history of diabetes, systolic blood pressure, total cholesterol and HDL cholesterol, and stratified by sex. Differences in risk ratios according to differing lengths of follow-up across studies was tested using meta-regression.

eFigure 9. Associations of NT-proBNP and HDL-cholesterol with fatal cardiovascular outcomes.

	No. of		Risk ratio (95% CI)	
	Cohorts	Events	Top vs. bottom third of NT-proBNP (■)	Bottom vs. top third of HDL-cholesterol (□)
**Individual outcomes**				
Fatal CHD	30	1945	2·33 (1·88, 2·89)	1·51 (1·28, 1·78)
Fatal stroke	28	764	2·27 (1·77, 2·92)	1·18 (0·96, 1·43)
Fatal heart failure	18	128	2·66 (1·35, 5·22)	1·34 (0·82, 2·18)
**Composite outcomes**				
Fatal CHD plus stroke	28	2635	2·51 (2·11, 2·98)	1·42 (1·21, 1·67)
Fatal CHD plus stroke plus heart failure	22	2108	2·45 (1·98, 3·03)	1·36 (1·14, 1·62)
**Exploratory outcome**				
Death due to other CVD*	27	938	3·32 (2·67, 4·12)	1·24 (1·04, 1·48)

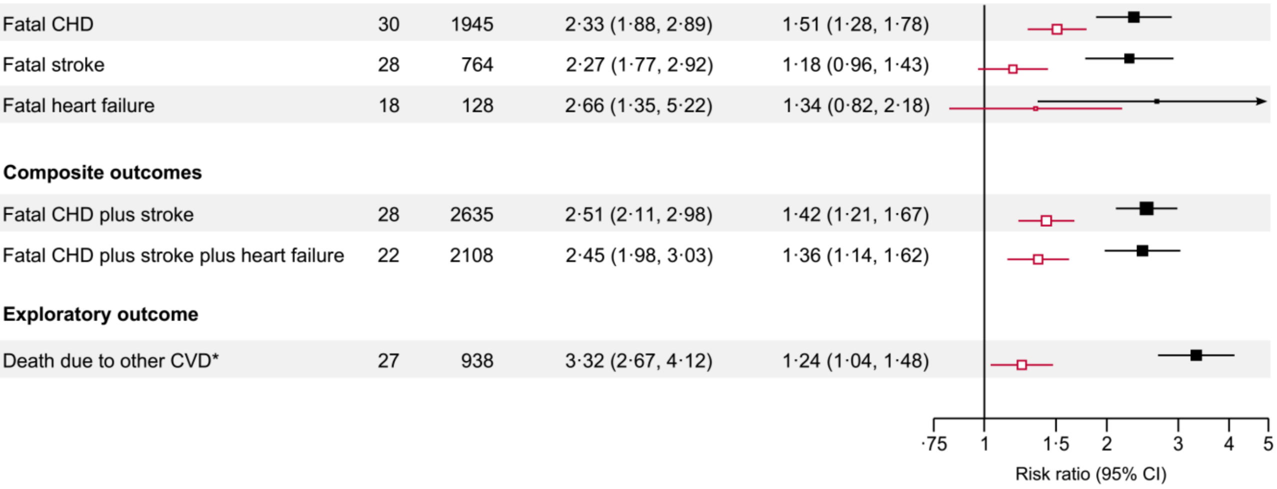

Analyses are <u>censored for fatal events only</u>. Risk ratios were adjusted for age, smoking status, history of diabetes, systolic blood pressure, total cholesterol and HDL-cholesterol (the latter only for the NT-proBNP analysis), and, where appropriate, stratified by sex. *Subsumes deaths due to cardiac arrhythmia, hypertensive disease, pulmonary embolism, ill-defined descriptions and complications of the heart, sudden death, aortic aneurysms, and peripheral vascular disease.

eFigure 10. Study-specific C-indices for the composite outcome of CHD plus stroke using conventional risk factors (including HDL-C) and corresponding changes after addition of information on NT-proBNP.

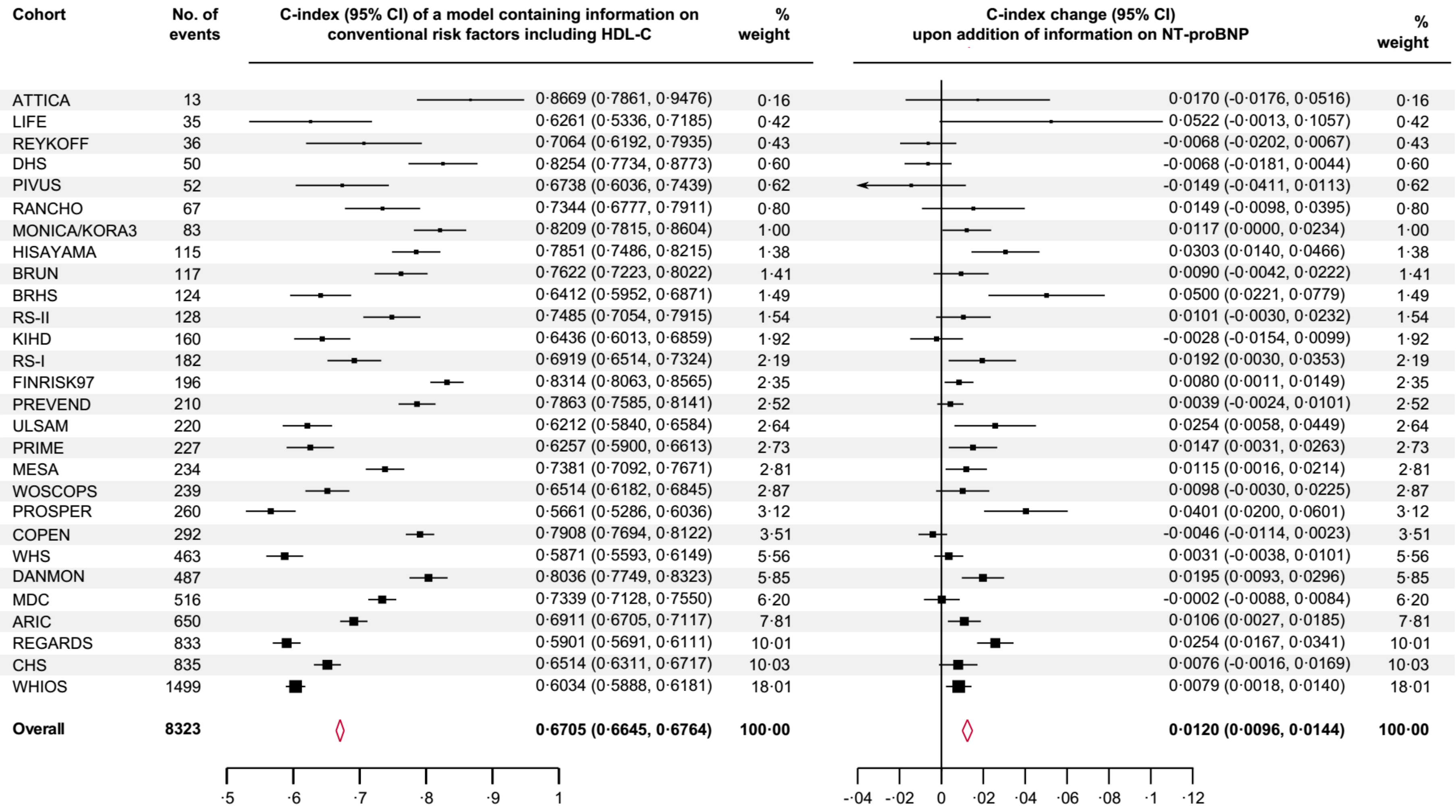

Full study names are listed in the **eAppendix 5**. CHS consists of the cohorts CHS1 and CHS2. DANMON consists of the cohorts 45yr1936, 70yr1914, GenMon, Monica2, and Monica3.

eFigure 11. Study-specific C-indices for the composite outcome of CHD plus stroke plus heart failure using conventional risk factors (including HDL-C) and corresponding changes after addition of information on NT-proBNP.

Cohort	No. of events	C-index (95% CI) of a model containing information on conventional risk factors including HDL-C	% weight	C-index change (95% CI) upon addition of information on NT-proBNP	% weight
LIFE	45	0·6278 (0·5414, 0·7141)	0·68	0·0635 (0·0101, 0·1169)	0·68
DHS	72	0·8066 (0·7645, 0·8487)	1·09	0·0226 (0·0050, 0·0402)	1·09
MONICA/KORA3	85	0·8155 (0·7742, 0·8569)	1·29	0·0112 (0·0003, 0·0221)	1·29
RANCHO	86	0·7328 (0·6818, 0·7838)	1·31	0·0237 (0·0017, 0·0458)	1·31
HISAYAMA	117	0·7950 (0·7601, 0·8299)	1·78	0·0254 (0·0109, 0·0399)	1·78
BRUN	126	0·7754 (0·7369, 0·8140)	1·91	0·0071 (-0·0063, 0·0204)	1·91
RS-II	136	0·7555 (0·7138, 0·7973)	2·07	0·0118 (-0·0023, 0·0259)	2·07
KIHD	161	0·6353 (0·5928, 0·6778)	2·45	-0·0039 (-0·0179, 0·0102)	2·45
RS-I	192	0·7048 (0·6668, 0·7428)	2·92	0·0156 (0·0001, 0·0311)	2·92
PRIME	228	0·6304 (0·5949, 0·6659)	3·46	0·0148 (-0·0006, 0·0303)	3·46
ULSAM	316	0·6242 (0·5929, 0·6556)	4·80	0·0430 (0·0210, 0·0651)	4·80
PROSPER	317	0·5595 (0·5262, 0·5929)	4·82	0·0631 (0·0407, 0·0855)	4·82
PREVEND	351	0·8024 (0·7815, 0·8234)	5·33	0·0156 (0·0085, 0·0228)	5·33
DANMON	543	0·8074 (0·7800, 0·8349)	8·25	0·0185 (0·0078, 0·0291)	8·25
MDC	599	0·7341 (0·7145, 0·7536)	9·10	0·0031 (-0·0066, 0·0128)	9·10
FINRISK97	653	0·7991 (0·7835, 0·8147)	9·92	0·0129 (0·0082, 0·0176)	9·92
CHS	1273	0·6682 (0·6521, 0·6843)	19·34	0·0185 (0·0097, 0·0274)	19·34
ARIC	1282	0·7053 (0·6912, 0·7195)	19·48	0·0184 (0·0117, 0·0250)	19·48
**Overall**	**6582**	**0·7148 (0·7084, 0.7211)**	**100·00**	**0·0190 (0·0159, 0·0222)**	**100·00**

Full study names are listed in the **eAppendix 5**. CHS consists of the cohorts CHS1 and CHS2. DANMON consists of the cohorts 45yr1936, 70yr1914, GenMon, Monica2, and Monica3.

eFigure 12. Improvement in risk discrimination of the composite outcome of CHD plus stroke plus heart failure by addition of information on NT-proBNP to conventional risk factors across clinically relevant subgroups.

Subgroup	No. of events	C-index, reference model	Change in C-index (95% CI)	P value
**Age at baseline**				
<60 years	951	0·7489	0·0120 (0·0059, 0·0181)	<0·0001
60-70 years	1423	0·6862	0·0297 (0·0208, 0·0387)	
>70 years	964	0·6694	0·0585 (0·0411, 0·0758)	
**Sex**				
Male	3049	0·7268	0·0212 (0·0165, 0·0259)	0·49
Female	2828	0·7375	0·0189 (0·0143, 0·0235)	
**Current smoker**				
No	4836	0·7235	0·0206 (0·0167, 0·0245)	1·00
Yes	1746	0·7140	0·0206 (0·0139, 0·0273)	
**Systolic blood pressure**				
Bottom third	1283	0·7142	0·0180 (0·0113, 0·0246)	0·017
Middle third	2039	0·7020	0·0246 (0·0175, 0·0316)	
Top third	3215	0·6793	0·0317 (0·0250, 0·0384)	
**History of diabetes**				
No	5405	0·7192	0·0206 (0·0170, 0·0242)	0·011
Yes	1177	0·6688	0·0376 (0·0250, 0·0503)	
**Total cholesterol**				
Bottom third	2000	0·7337	0·0304 (0·0235, 0·0373)	0·0027
Middle third	2307	0·7249	0·0163 (0·0116, 0·0210)	
Top third	2275	0·7041	0·0173 (0·0115, 0·0230)	
**HDL-cholesterol**				
Bottom third	2645	0·7038	0·0218 (0·0165, 0·0271)	0·72
Middle third	2158	0·7249	0·0185 (0·0126, 0·0244)	
Top third	1779	0·7274	0·0209 (0·0143, 0·0276)	
**Body mass index**				
Bottom third	1642	0·7318	0·0205 (0·0141, 0·0270)	0·80
Middle third	1961	0·7235	0·0192 (0·0135, 0·0248)	
Top third	2567	0·7134	0·0176 (0·0121, 0·0232)	
**C-reactive protein**				
Bottom third	1269	0·7102	0·0153 (0·0093, 0·0212)	0·11
Middle third	1829	0·7038	0·0231 (0·0164, 0·0299)	
Top third	2394	0·7035	0·0236 (0·0173, 0·0298)	
**Estimated GFR**				
<60 ml/min/1.73m^2	761	0·7061	0·0309 (0·0188, 0·0429)	0·064
60-90 ml/min/1.73m^2	1945	0·7031	0·0226 (0·0156, 0·0295)	
>90 ml/min/1.73m^2	1254	0·7204	0·0151 (0·0081, 0·0221)	
**Statin use**				
No	944	0·7634	0·0268 (0·0175, 0·0361)	0·43
Yes	98	0·7307	0·0413 (0·0061, 0·0765)	
**Use of anti-hypertensives**				
No	1898	0·7155	0·0127 (0·0063, 0·0192)	0·0069
Yes	1239	0·6772	0·0274 (0·0189, 0·0359)	
**Study design**				
Cohort	6039	0·7255	0·0191 (0·0158, 0·0224)	0·52
Case-cohort	543	0·8259	0·0185 (0·0078, 0·0291)	

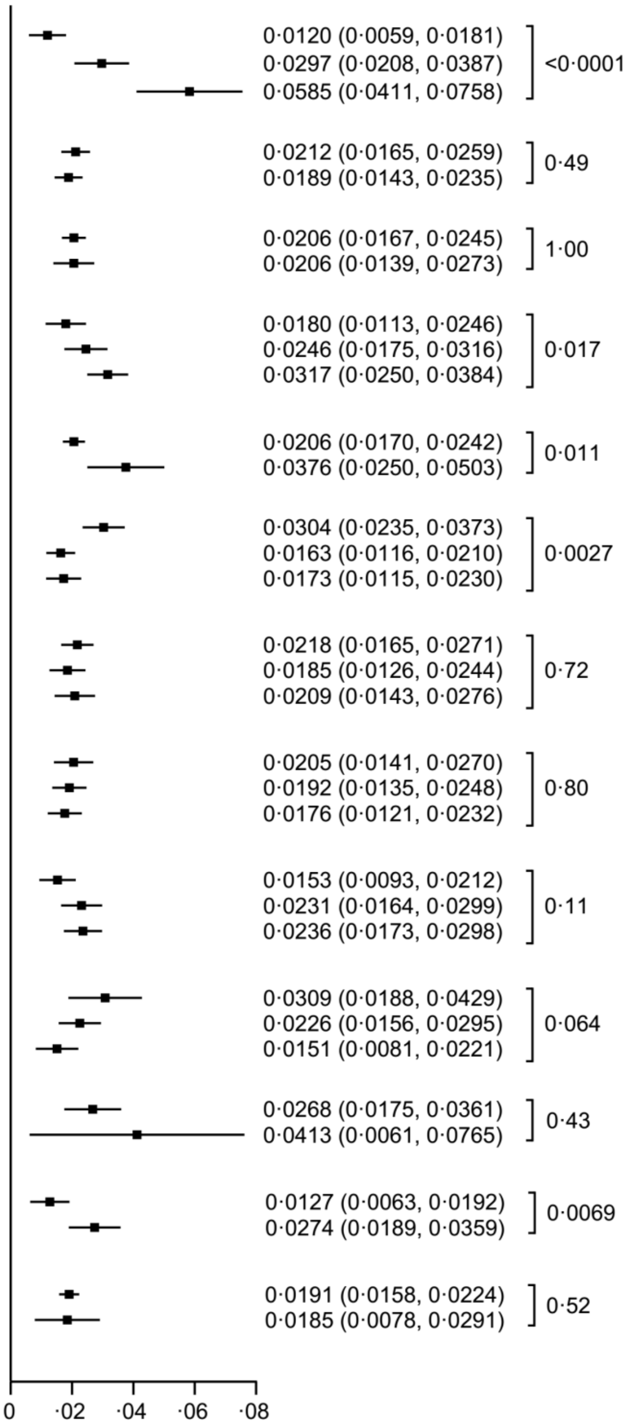

The base model included information on the conventional risk factors age, smoking, systolic blood pressure, history of diabetes, and levels of total and HDL-cholesterol, and was stratified by study and sex. NT-proBNP concentration was log-transformed and modeled using both linear and quadratic terms. χ^2 tests were used to test for differences in changes in discrimination measures across subgroups. Abbreviations: estimated GFR, estimated glomerular filtration rate (using the CKD-EPI formula 2009).

eFigure 13. Improvement in risk discrimination of individual and composite cardiovascular outcomes by addition of information on NT-proBNP to a model with information on conventional risk factors and a model with information on conventional risk factors, ethnicity, and anti-hypertensive medication.

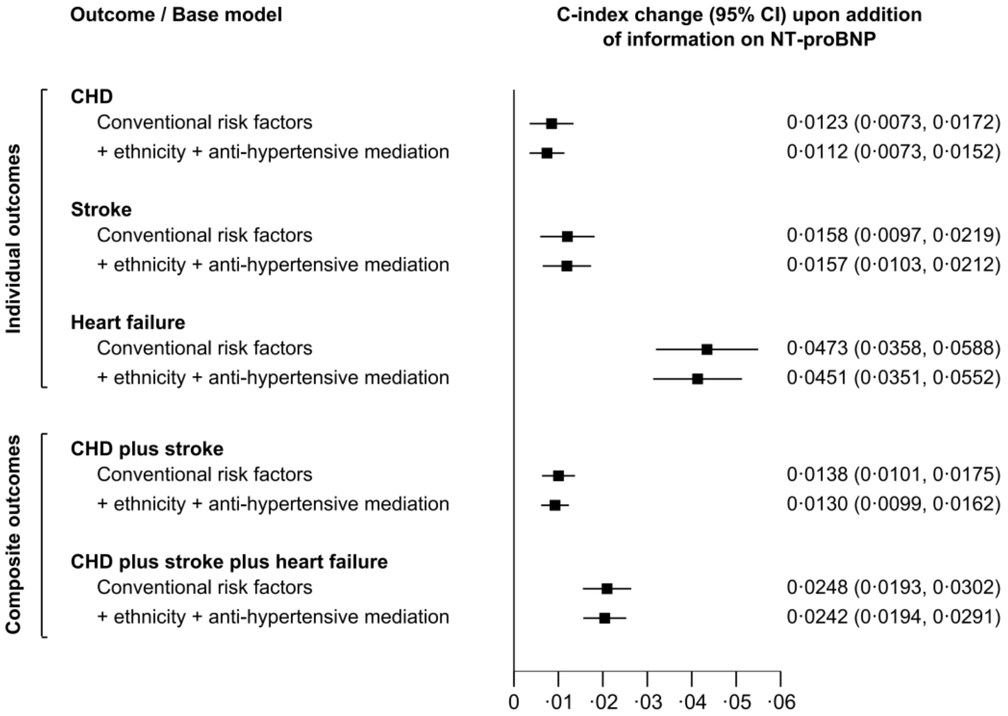

The analyses involved the following number of outcomes: 2772 for CHD (from 19 cohorts), 2534 for stroke (from 18 cohorts), 1264 for heart failure (from 10 cohorts), 5,306 for the composite outcome of CHD plus stroke (from 19 cohorts), and 3482 for the composite outcome of CHD plus stroke plus heart failure (from 13 cohorts). Conventional risk factors: age, sex, smoking, systolic blood pressure, history of diabetes, and levels of total and HDL-cholesterol. NT-proBNP concentration was log-transformed and modeled using both linear and quadratic terms.

eFigure 14. Sensitivity analysis of the improvement in risk discrimination of individual and composite cardiovascular outcomes by addition of information on NT-proBNP to conventional risk factors.

	Outcome / Analysis type	No. of cohorts	No. of events	C-index change (95% CI)
Individual outcomes	**CHD**			
	Primary analysis	32	4552	0·0121 (0·0088, 0·0154)
	Using a cut-off value for NT-proBNP of 100 pg/ml	32	4552	0·0052 (0·0027, 0·0078)
	Excluding people with high baseline NT-proBNP	32	4429	0·0086 (0·0056, 0·0116)
	Studies with fatal and non-fatal outcomes	26	4236	0·0129 (0·0093, 0·0165)
	**Stroke**			
	Primary analysis	30	3768	0·0114 (0·0076, 0·0152)
	Using a cut-off value for NT-proBNP of 100 pg/ml	30	3768	0·0054 (0·0026, 0·0083)
	Excluding people with high baseline NT-proBNP	30	3670	0·0092 (0·0056, 0·0129)
	Studies with fatal and non-fatal outcomes	23	3076	0·0131 (0·0085, 0·0177)
	**Heart failure**			
	Primary analysis	16	2021	0·0378 (0·0302, 0·0454)
	Using a cut-off value for NT-proBNP of 100 pg/ml	16	2021	0·0223 (0·0161, 0·0284)
	Excluding people with high baseline NT-proBNP	15	1911	0·0284 (0·0213, 0·0355)
	Studies with fatal and non-fatal outcomes	9	852	0·0329 (0·0226, 0·0432)
Composite outcomes	**CHD plus stroke**			
	Primary analysis	28	8323	0·0120 (0·0096, 0·0144)
	Using a cut-off value for NT-proBNP of 100 pg/ml	28	8323	0·0058 (0·0039, 0·0076)
	Excluding people with high baseline NT-proBNP	28	8102	0·0093 (0·0071, 0·0116)
	Studies with fatal and non-fatal outcomes	24	7930	0·0129 (0·0103, 0·0155)
	**CHD plus stroke plus heart failure**			
	Primary analysis	18	6582	0·0190 (0·0159, 0·0222)
	Using a cut-off value for NT-proBNP of 100 pg/ml	18	6582	0·0101 (0·0076, 0·0127)
	Excluding people with high baseline NT-proBNP	18	6346	0·0143 (0·0114, 0·0172)
	Studies with fatal and non-fatal outcomes	18	6582	0·0190 (0·0159, 0·0222)

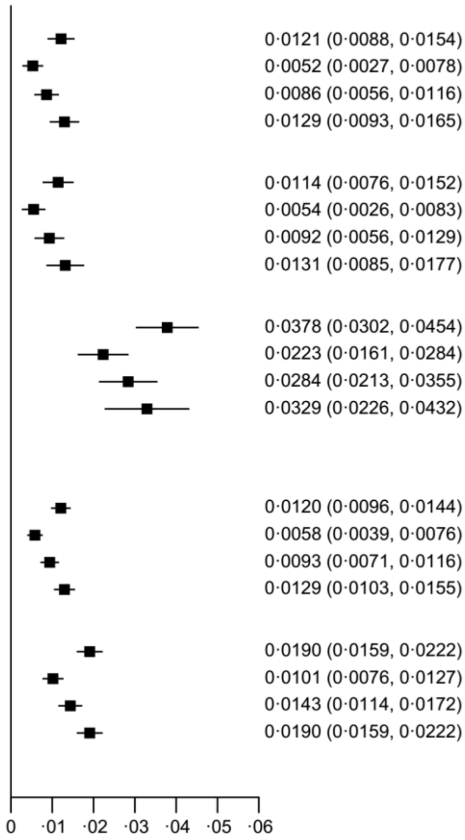

The base model included information on the conventional risk factors age, smoking, systolic blood pressure, history of diabetes, and levels of total and HDL-cholesterol, and was stratified by study and sex. NT-proBNP concentration was log-transformed and modeled using both linear and quadratic terms. The cutoff used to exclude people with high baseline levels of NT-proBNP were: >450 pg/ml for people aged 50 years or younger, >900 pg/ml for people aged 50-75 years, and >1800 pg/ml for people aged 75 years or older.
